# 1,2,4-Oxadiazole Derivatives: Physicochemical Properties, Antileishmanial Potential, Docking and Molecular Dynamic Simulations of *Leishmania infantum* Target Proteins

**DOI:** 10.3390/molecules29194654

**Published:** 2024-09-30

**Authors:** Deyzi C. S. Barbosa, Vanderlan N. Holanda, Elton M. A. Lima, Marton K. A. Cavalcante, Maria Carolina A. Brelaz-de-Castro, Elton J. F. Chaves, Gerd B. Rocha, Carla J. O. Silva, Ronaldo N. Oliveira, Regina C. B. Q. Figueiredo

**Affiliations:** 1 Department of Microbiology, Aggeu Magalhães Institute (IAM-FIOCRUZ), Recife 50740-465, PE, Brazil; deyzi-caroline@hotmail.com; 2 Department of Biomedicine, University Center of Vitória de Santo Antão (UNIVISA), Vitória de Santo Antão 55610-050, PE, Brazil; 3 Center for Exact and Natural Sciences, Federal University of Pernambuco (UFPE), Recife 50740-560, PE, Brazil; 4 Parasitology Laboratory, Academic Center of Vitória, Federal University of Pernambuco (UFPE), Recife 50670-420, PE, Brazil; 5 Department of Immunology, Aggeu Magalhães Institute (IAM-FIOCRUZ), Recife 50740-465, PE, Brazil; 6 Department of Chemistry, Federal University of Paraíba (UFPB), João Pessoa 58051-900, PB, Brazil; 7 Department of Fundamental Chemistry, Federal University of Pernambuco (UFPE), Recife 50740-540, PE, Brazil; cjasmine0803@gmail.com; 8 Department of Chemistry, Federal Rural University of Pernambuco (UFRPE), Recife 52171-900, PE, Brazil; ronaldo.noliveira@ufrpe.br

**Keywords:** visceral leishmaniasis, chemotherapy, 1,2,4-oxadiazole, ultrastructure, molecular docking, molecular dynamic

## Abstract

Visceral leishmaniasis (VL), caused by protozoa of the genus *Leishmania*, remains a significant public health concern due to its potentially lethal nature if untreated. Current chemotherapy options are limited by severe toxicity and drug resistance. Derivatives of 1,2,4-oxadiazole have emerged as promising drug candidates due to their broad biological activity. This study investigated the effects of novel 1,2,4-oxadiazole derivatives (**Ox1**–**Ox7**) on *Leishmania infantum*, the etiological agent of VL. In silico predictions using SwissADME suggest that these compounds have high oral absorption and good bioavailability. Among them, **Ox1** showed the most promise, with higher selectivity against promastigotes and lower cytotoxicity towards L929 fibroblasts and J774.G8 macrophages. **Ox1** exhibited selectivity indices of 18.7 and 61.7 against *L. infantum* promastigotes and amastigotes, respectively, compared to peritoneal macrophages. Ultrastructural analyses revealed severe morphological damage in both parasite forms, leading to cell death. Additionally, **Ox1** decreased the mitochondrial membrane potential in promastigotes, as shown by flow cytometry. Molecular docking and dynamic simulations indicated a strong affinity of **Ox1** for the *L. infantum* CYP51 enzyme. Overall, **Ox1** is a promising and effective compound against *L. infantum*.

## 1. Introduction

Visceral leishmaniasis (VL), caused by protozoa of the genus *Leishmania*, is a neglected tropical disease and represents the most severe form of leishmaniasis. Two species, *Leishmania donovani* and *Leishmania infantum* (syn. *L. chagasi*), are identified as responsible for causing VL in the Old and New World, respectively [1]. VL is considered a systemic infection, causing hyperplasia of the reticuloendothelial system and infiltrating organs such as the spleen, liver, bone marrow, and lymph nodes [2,3]. Without proper treatment, VL can cause death in over 95% of cases due to complications, including organ dysfunction, immune suppression, hematological abnormalities, increased susceptibility to secondary infections, and overall debilitation [4,5].

Currently, no prophylactic vaccine is available for human leishmaniasis [6]. Consequently, the existing treatment relies on drugs such as pentavalent antimonials (sodium stibogluconate and meglumine antimoniate), Amphotericin B, paromomycin, and miltefosine [7]. However, these drugs present several drawbacks, including prolonged treatment, toxicity, and adverse effects such as nausea, vomiting, arthralgia, pancreatitis, cardiac arrhythmias, and hepatotoxicity. Additionally, they are associated with high costs and the emergence of resistant strains [8,9]. Therefore, the development of new chemotherapeutic alternatives that are cost-effective and easily and quickly obtainable for the treatment of visceral leishmaniasis remains imperative [10].

Nitrogenated-based heterocyclic compounds, such as 1,2,4-Oxadiazole-derived molecules show promising biological activity, largely due to the hydrophilic and electron-donating properties of the oxadiazole ring [11], which enable binding with enzymes and other biomolecules, via non-covalent interactions [12,13,14]. Additionally, the thermal and chemical resistance of the oxadiazole ring contributes to its metabolic stability in biological systems [15]. 1,2,4-Oxadiazole is a five-membered ring composed of one oxygen atom, two nitrogen atoms, and two carbons, with significant applications in medicinal chemistry [16]. This pharmacophoric group, first identified as a natural product isolated from the mollusk *Phidiana militaris*, has demonstrated high toxicity against tumoral and non-tumoral mammalian cells [17]. Since then, investigations on 1,2,4-oxadiazole derivatives have demonstrated various bioactive properties, including anticancer [18], antitubercular [19,20], anti-inflammatory [21], antibacterial [22], and antiparasitic effects [23,24]. Furthermore, clinically used drugs, such as Oxolamine and Irrigor for cough treatment (antitussive) and vasodilatation agent, respectively, contain a 1,2,4-oxadiazole moiety in their molecular structure, highlighting its significance in drug design and discovery [25,26].

Recently, Pinheiro et al. [27] reported the use of a compound containing 1,2,4-oxadiazole (N-cyclohexyl-1,2,4-oxadiazole) against the promastigote form of *L. infantum*, rendering this group promising to combat Leishmaniasis. In this regard, the need for the development of safe, low-cost, and highly selective therapeutic agents incorporating 1,2,4-oxadiazole prompted us to investigate the potential of seven 1,2,4-oxadiazole derivatives as potential chemotherapeutic agents against *L. infantum*. Together, our data highlight the compound **Ox1** as a candidate for further development as an anti-*leishmania* drug due to its higher selectivity for *L. infantum*.

## 2. Results and Discussion

### 2.1. Chemistry

**Scheme 1:**
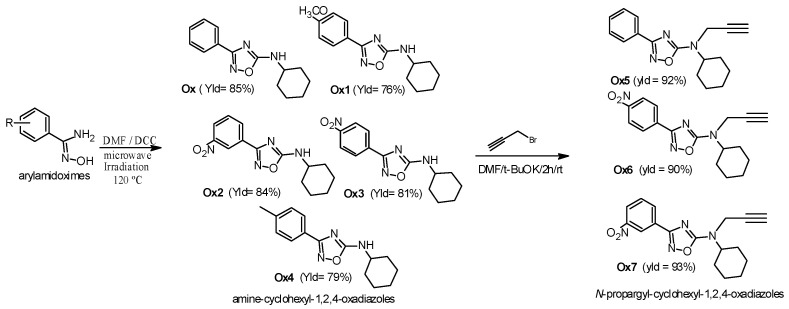
Representative scheme of the synthesis of 1,2,4-oxadiazole derivatives **Ox1**–**Ox7**.

In the pursuit of new alternative compounds for treating Leishmaniasis, it is imperative to focus on synthesizing safer, more effective, and economically sustainable molecules against the parasite, especially in the context of neglected diseases. In previous work, Oliveira et al. [28] described an alternative protocol to the classical methods used to synthetize 1,2,4-oxadiazole derivatives, employing focused microwave irradiation (FMWI), a green chemistry method characterized by its low cost and reduced time consumption [29]. Using this approach, 1,2,4-oxadiazole derivatives **Ox1**, **Ox2**, **Ox3**, and **Ox4** were obtained in high yields from 76 to 85% (Scheme 1). Therefore, we extended the synthesis of other series to explore the activity of *N*-alkylated derivatives. Propargyl-amino derivatives have recently shown broad biological activity, for instance, against lung cancer [20], Alzheimer’s [30], Parkinson’s diseases [31], and anti-mycobacterial agents [32]. *N*-propargyl derivatives were synthesized from the corresponding 3-aryl-5-amine-cyclohexyl-1,2,4-oxadiazoles. For this, we selected three amine-cyclohexyl-1,2,4-oxadiazoles to react with propargyl bromide, using *tert*-BuOK in DMF for 2 h under room temperature to furnish the *N*-alkylated **Ox5**, **Ox6**, and **Ox7** in yields up to 90% (Scheme 1).

### 2.2. Physicochemical Properties

The compounds **Ox1**–**Ox7** were investigated for their physicochemical properties, cytotoxic, and leishmanicidal properties (Table 1 and Table 2). It is widely recognized that the existing drugs available for the treatment of Leishmaniasis pose challenges due to their poor solubility, requiring parenteral administration [33]. While miltefosine stands as the sole oral drug available for leishmaniasis, its extensive use in large-scale treatment programs is hindered by its prohibitive cost. Furthermore, an increase in miltefosine-resistant parasites has been reported [34,35]. In this regard, predicting the physicochemical properties of potential drug candidates for leishmaniasis treatment becomes pivotal, providing crucial insights during the initial phases of drug discovery. Herein, we used SwissADME descriptors to analyze the drug-likeness of **Ox1**–**Ox7** based on Lipinski’s and Veber’s rules, which consider relevant physicochemical parameters related to the druggability for oral drugs [36]. Lipinski´s rule of five states that, for good oral bioavailability, a drug candidate should not violate more than two parameters: (1) molecular weight (MW) ≤ 500 g/mol; (2) number of hydrogen bond acceptors (HBA) ≤ 10; (3) number of hydrogen bond donors (HBD) ≤ 5; (4) and octanol–water partition coefficient (LogP) < 5.

The molecular weights of all derivatives (**Ox1**–**Ox7**) are within the acceptable range of ≤500 g/mol, ranging from 257.33 g/mol (**Ox4**) to 326.35 g/mol (**Ox6** and **Ox7**). These compounds exhibited HBA and HBD values below 10 and 5, respectively, as well as LogP < 5. Furthermore, the compounds exhibited n-ROTB values ≤ 10 and TPSA values below 140 Å^2^. As proposed by Veber et al. [37], the parameters n-ROTB and TPSA are effective in predicting oral bioavailability. The n-ROTB parameter is associated with the compound’s flexibility and interaction with other molecules, while TPSA is associated with drug absorption through biological cell membranes. The predicted drug likenesses of **Ox1**–**Ox7** were compared with the standard drug miltefosine, which follows Lipinski’s rules but violates the n-ROTB established by Veber, with a value of n-ROTB = 20. Previous studies have shown that as a molecule becomes more flexible (n-ROTB > 10), its oral bioavailability decreases, directly impacting its biological activity [37,38,39].

### 2.3. Cytotoxicity in Mammalian Cells and Effects of ***Ox1**–**Ox7*** on L. infantum Promastigotes

Since the **Ox1**–**Ox7** compounds demonstrated favorable drug-likeness properties for potential oral formulations, we investigated their in vitro effects on the viability of mammalian cells. Initially, the cytotoxic potential of the compounds was evaluated on L929 fibroblast and J774.G8 macrophage lineages. Although it is well known that the macrophages and other phagocytic cells were the main targets of *Leishmania* infection, it has been reported that non-professional phagocytic cells such as epithelial cells, hepatocytes, and fibroblasts can also serve as non-canonical host cells [40]. For instance, many studies point to fibroblasts as already established host cells that are important in latent leishmaniasis, facilitating the differentiation and proliferation of amastigotes, regardless of the *Leishmania* species involved [41,42].

Our results showed that all the compounds exhibited moderate toxicity, with CC_50_ ranging from 290 µM (**Ox4**) to 375 µM (**Ox5**) for fibroblasts and from 160 µM (**Ox5**) to 293.1 µM (**Ox1**) for macrophages. For the compounds **Ox7** (fibroblasts), **Ox3**, and **Ox4** (macrophages), it was not possible to estimate the CC_50_ at the tested concentrations, thus the CC_50_ was assumed to be >200 µM (Table 2, Figures S1 and S2). An initial screening of the in vitro antileishmanial activity of **Ox1**–**Ox7** was conducted on *L. infantum* promastigotes. Overall, all the compounds exhibited greater cytotoxicity towards this developmental stage of the parasite compared to mammalian cells. The inhibitory concentration by 50% (IC_50_) for promastigotes ranged from 32.9 µM (**Ox1**) to 336 µM (**Ox3**) (Figure S3).

**Figure 1:**
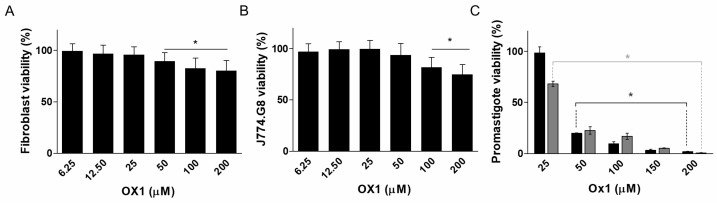
Representative graphs of the effects of **Ox1** on mammalian cells and promastigotes. (**A**) Activity of **Ox1** on the viability of fibroblast (L929) and (**B**) macrophages (J774.G8) after 48 h of drug treatment. (**C**) Effects of **Ox1** on promastigotes after 24 (black bars) and 48 (gray bars) hours of treatment. Values represent the mean ± standard deviation of three independent experiments in triplicate. * Significant differences at *p* < 0.05 compared to untreated control.

When comparing the cytotoxicity towards mammalian cells with the leishmanicidal activity of the compounds, we observed that **Ox1** showed the highest selectivity against promastigotes compared to fibroblasts and macrophages, with a selectivity index (SI) of 9.7 and 8.9, respectively. Notably, only at concentrations up to 50 µM for fibroblasts and 100 µM for J774.G8, the treatment with **Ox1** led to a significant decrease in cell viability. At the highest tested concentration (200 µM), a reduction of cell viability by 20% for fibroblasts and 26% for macrophages was observed (Figure 1A,B). In contrast, a significant decrease in promastigote viability was observed at concentrations up to 25 µM (48 h) and 50 µM (24 h) of treatment (Figure 1C).

The treatment of promastigote with **Ox1**, for 24 h, reduced the growth of parasites by 66% at 50 µM. In cells treated for 48 h, a 32% reduction in promastigote viability was observed earlier at 25 µM. At the highest concentration tested (200 µM), promastigote viability inhibition exceeded 90% regardless of the cultivation time. The activity of **Ox1** on promastigote can be due to the presence of both 4-methoxy and phenyl associated with a 1,2,4-oxadiazole backbone (Scheme 1).

Compounds **Ox2**, **Ox5**, and **Ox6** exhibited a SI lower than 5.0 and were therefore not considered for additional studies, along with compounds **Ox7**, **Ox3**, and **Ox4**, for which estimating the SI was not possible for at least one of the evaluated cell lines. Due to its higher selectivity index towards promastigote forms and lower cytotoxicity towards fibroblasts and J774.G8 macrophages (Figure 1), **Ox1** was chosen for further investigation on its putative mechanisms of action in promastigotes and their effects on intracellular amastigote forms.

The structure–activity relationship (SAR) of the 3-aryl-5-amine-cyclohexyl-1,2,4-oxadiazole series revealed that the presence of a methoxy (electron-donating group) at the *p*-position in the phenyl moiety increased the activity of compound **Ox1** (IC_50_ = 32.9 µM) by about 10 times when compared to compound **Ox3** (*p*-NO_2_, an electron-withdrawing group) with IC_50_ = 336 µM. In general, the insertion of the propargyl group at the nitrogen atom (see **Ox6** and **Ox7**) induced a moderate increase in the activity, with IC_50_ values of 92.2 and 98.2 µM, respectively, compared to compounds **Ox2** (IC_50_ = 220 µM) and **Ox3** (IC_50_ = 336 µM). Despite these better results, *N*-propargylated derivatives showed SI < 5. Then, compound **Ox1** still remains the best compound with antileishmanial activity. These data suggest that the NH moiety and the OCH_3_ group appear to be crucial for the leishmanicidal activity, likely due to the electronic interaction between the donor and acceptor fragments [43], these findings will be further explained with docking studies in Section 2.8.

### 2.4. The Effects of ***Ox1*** on L. infantum Amastigotes

**Figure 2:**
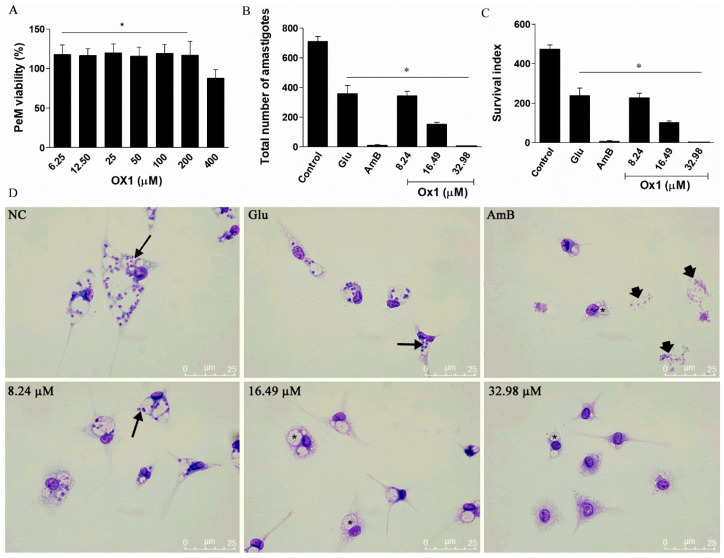
Effects of **Ox1** on the viability of PeMs and intracellular amastigotes of forms of *L. infantum*. (**A**) Percentage of viable treated PeMs compared to untreated ones. (**B**) Total number of amastigotes in 300 infected cells. (**C**) Survival index (SuI) of amastigotes in PeMs. Glu—glucantime; AmB—Amphotericin B. Each bar represents the mean ± SD of three independent experiments performed in duplicate. * Significant differences at *p* < 0.05 compared to the untreated control. (**D**) Representative images of untreated infected-PeMs negative control (NC) or treated with Glu, AmB, or **Ox1** at ¼ (8.24 µM), ½ IC_50_ (16.42 µM), and IC_50_ (32.98 µM) for promastigote forms. Intracellular amastigotes are indicated by arrows. Note in the culture treated with AmB the presence of cellular debris (thick arrow). Cells treated with IC_50_ and ½ IC_50_ of **Ox1** or AmB, presenting empty parasitophorous vacuoles (*) can be also observed. Bars 25 µm.

To study the effects of **Ox1** on intracellular amastigotes, we selected peritoneal macrophages from BALB/c mice (PeM) as our infection model due to their similarity to primary tissue macrophages, as opposed to cell line-derived ones [44] (Figure 2A). Our results showed that the viability of macrophages was significantly increased at concentrations ranging from 6.25 to 200 µM. This discrete but statistically significant enhancement in cell viability suggested that at these concentrations, **Ox1** might exert a cytoprotective effect on peritoneal macrophages. Only at 400 µM, the viability of PeMs was reduced by 13% (Figure 2A) compared to the control cells. The CC_50_ for PeMs was estimated to be 617 µM, which was higher than the values found for J774.G8 macrophages and fibroblasts.

The treatment of infected PeMs with **Ox1** significantly decreased both the total number of parasites in infected cells (Figure 2B) and the survival index (SuI) of amastigotes inside the macrophages (Figure 2C). The inhibitory effect of **Ox1** on amastigotes was comparable to glucantime at 346.19 µM and Amphotericin B at 10.55 µM, (Figure 2B,C). The IC_50_ value for the amastigote forms of *L. infantum* in PeMs was estimated to be 10 µM. It has been reported that drugs presenting an SI ≥ 10 are considered promising candidates for advancing in preclinical studies toward the development of new drugs against leishmaniasis [45,46]. Interestingly, the SI of **Ox1** against amastigotes (61.7) was higher than that found for promastigotes (18.7). This could be attributed, in part, to the lower cytotoxic effect of this drug on PeMs (CC_50_ = 617 µM), thus elevating the selectivity index value. These results suggest that, in addition to its direct activity on amastigotes, **Ox1** may also enhance macrophage function in combating the infection. The improved viability of **Ox1**-treated macrophages supports this hypothesis. The inhibitory effect of **Ox1** on intracellular macrophages can be better visualized in Figure 2D. Untreated infected macrophages, used as negative control (NC) presented large parasitophorous vacuoles containing several amastigotes. The treatment with the reference drug glucantime (Glu) or Amphotericin B (AmB) reduced the number of intracellular amastigotes in PeMs. However, the presence of cellular debris and the decrease in PeMs/field, in cultures treated with AmB, clearly demonstrated the cytotoxic effects of this drug on PeMs. The treatment of infected cells with **Ox1** significantly reduced the number of intracellular amastigotes at ½ IC_50_ (8.24 µM) and decreased the number of infected cells at the higher concentrations tested (16.49 and 32.98 µM).

### 2.5. Effects of ***Ox1*** on Ultrastructure of L. infantum Promastigotes

**Figure 3:**
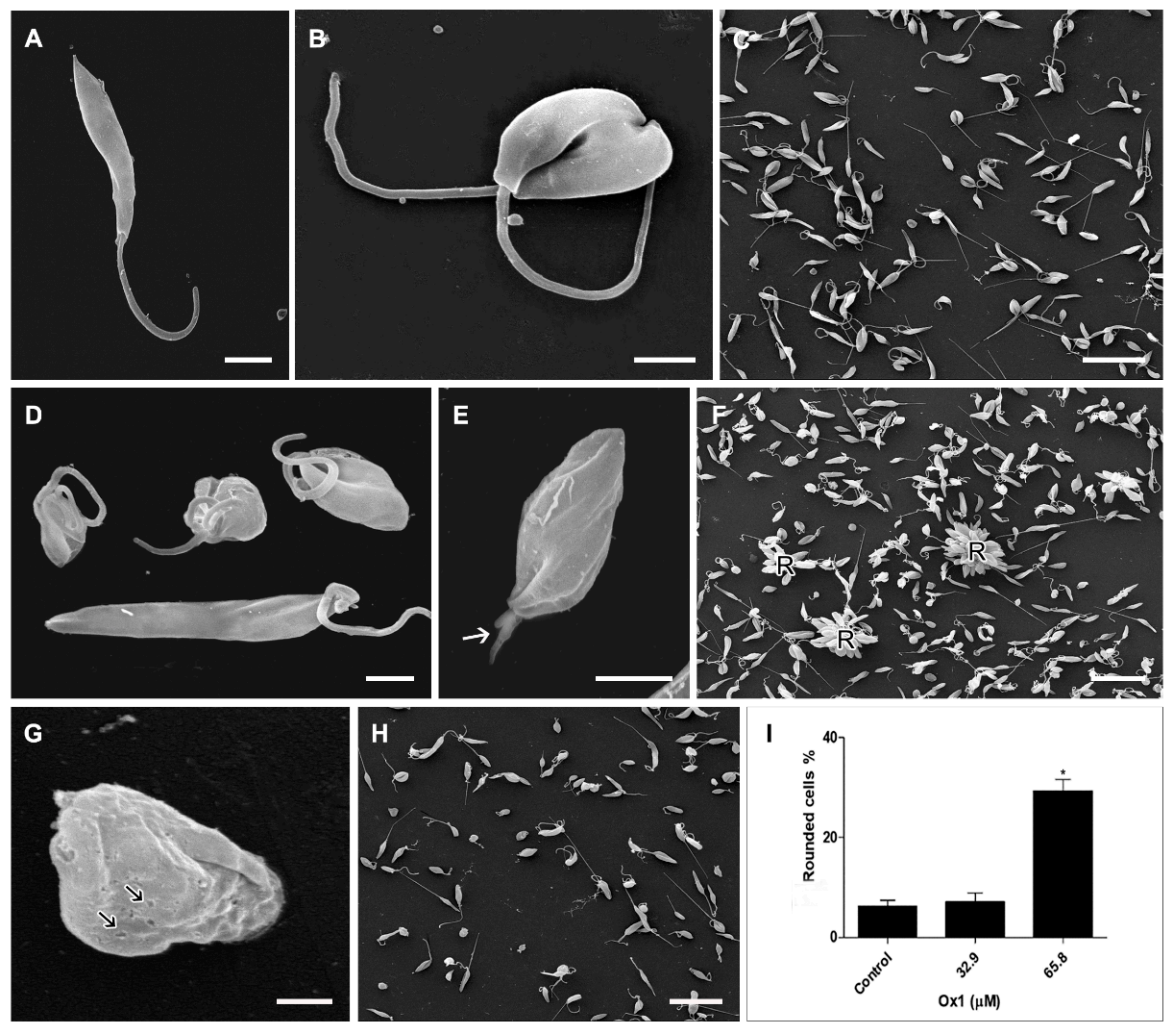
Scanning electron microscopy of control and treated promastigotes of *L. infantum*. (**A**) Detail of untreated promastigote showing elongated cell body, smooth plasma membrane, and long flagellum. (**B**) Untreated dividing promastigotes showing a preserved morphology. (**C**) Low magnification of untreated culture showing the predominance of elongated and dividing cells. (**D**) treated culture showing rounded cells with twisted flagellum around the parasite cell body next to an elongated cell. (**E**) High magnification of treated cell showing rounded cell body, septation, and altered and short flagellum (arrow). (**F**) Low magnification of culture treated with IC_50_ **Ox1** showing numerous promastigote rosettes (R). (**G**) Severe injured cells treated with 2× IC_50_ **Ox1**. Note the presence of cell membrane perforations (arrow), the loss of characteristic morphology, and the absence of visible flagellum. (**H**) At low magnification, it is possible to observe the decrease in cell number and the increase in cells presenting altered rounded morphology. (**I**) Representative graph of the percentage of rounded promastigotes treated with IC_50_ (32.98 µM) and 2× IC_50_ (65.96 µM). A total of 500 cells were randomly counted per sample. * Significant differences at *p* < 0.05 compared to the untreated control. Bars: (**A**,**B**,**D**,**E**) =2 µm; (**C**,**F**,**H**) =20 µm; (**G**) =1 µm.

To further investigate the effects of **Ox1** on the ultrastructure and morphology of promastigotes, transmission electron microscopy (TEM) and scanning electron microscopy (SEM) were performed on both treated and non-treated parasites. SEM images of untreated parasites (Figure 3A,B) revealed promastigotes exhibiting well-preserved morphology with a slender cell body, a smooth and intact cell surface, and elongated free flagellum. At low magnification, it is possible to observe the general aspect of promastigote culture showing preserved promastigotes and a high number of dividing cells (Figure 3C). On the other hand, the treatment of parasites with **Ox1** at both 32.98 μM (IC_50_) (Figure 3D–F) and 65.96 μM (2× IC_50_) (Figure 3G–I) induced drastic morphological changes compared to the control cells. The most prominent morphological alterations observed, mainly at low concentrations of **Ox1**, were the shortening and rounding of cell bodies and the coiling of the flagellum around the parasite’s body, occasionally merging with the plasma membrane (Figure 3D,E). Although the percentage of rounding cells in control (5%) was comparable to the parasites treated with the IC_50_ (7%), at twice the IC_50_ rounding cells corresponds to 30% of the total of cells (Figure 3I). At low magnification, we also observed an increased number of promastigote rosettes (Figure 3F). It has been shown that individual promastigotes can associate with each other forming clusters of different sizes in the culture and inside the phlebotomine vector [47]. The formation of rosettes has been associated with starvation, delayed multiplication, a mating strategy, or even an in vitro artifact [48]. However, we cannot dismiss the possibility that the presence of these clusters in treated cultures may represent an adaptive response of the protozoa to the stress induced by the treatment. Conversely, at higher concentrations of **Ox1**, the deleterious effects of the drug outweigh the parasites’ ability to cope with the induced stress, leading to the absence of rosettes, which are no longer observed (Figure 3H). Parasites displaying septation of the cell body with short or absent flagellum were mainly observed in cells treated with IC_50_ of **Ox1** (Figure 3E). The septation of the cell body in trypanosomatids, including *Leishmania* spp. has been linked to the effects of various drugs on cellular division, mainly those that inhibit the biosynthesis of ergosterol, thereby impairing de novo membrane synthesis and cytokinesis [49]. This disruption can cause incomplete division and septation of the cell body. At higher concentrations of **Ox1**, it is possible to observe damage to the cell surface in rounded-shaped promastigotes and the absence of an evident flagellum (Figure 3G,H).

The flagellum of Kinetoplastida parasites, like those belonging to the *Leishmania* and *Trypanosoma* genus, is essential for establishing and sustaining infections in hosts. Apart from its primary role in parasite motility, recent studies have identified additional functions such as attachment to host membranes in both the insect vector and mammalian host, interactions between parasites including mating and social motility, the secretion of vesicles, and sensation [50,51]. In this regard, the effects of **Ox1** may not only compromise the parasite motility but also the other functions in which the flagellum is involved. The shortening of the flagellum in *Leishmania* spp. has been reported in response to drugs that induce oxidative stress, disturbances in the cytoskeleton and/or its associated proteins, and disrupted mitochondrial energetic metabolism in this parasite [52,53,54].

**Figure 4:**
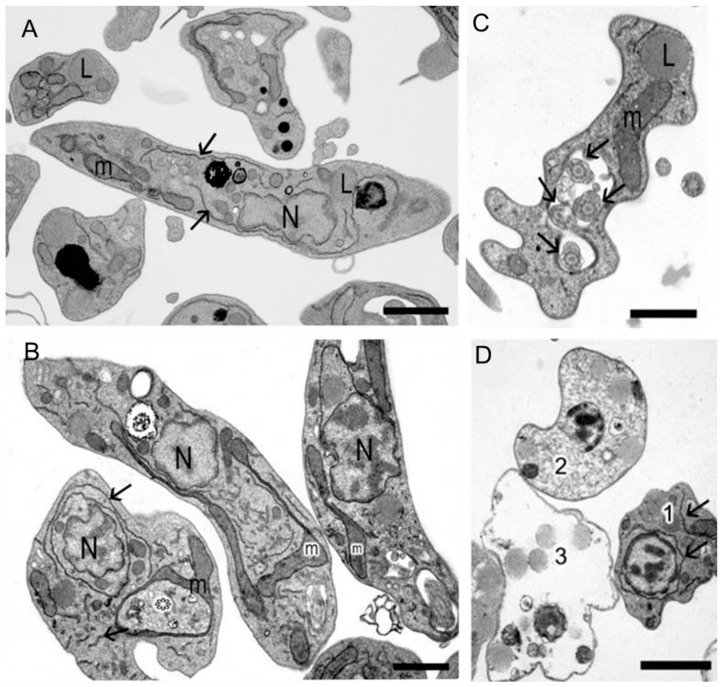
Transmission electron microscopy of control and treated promastigotes. (**A**) Untreated promastigote showing dispersed endoplasmic reticulum (arrow), preserved nucleus (N), mitochondrion (m), and lipid droplets (L). (**B**) Promastigote treated with IC_50_ **Ox1** showing altered mitochondrion (asterisk) and increased endoplasmic reticulum (arrow). (**C**) Promastigote treated with 2× IC_50_ showing multiple flagella within the flagellar pocket (arrow). (**D**) Note the presence of varying levels of cellular damage (1–3), increase in endoplasmic reticulum (1), and lipid droplets (2 and 3). Bars: (**A**,**B**)—2 µm. (**C**,**D**)—1 µm.

The detrimental effects of **Ox1** on promastigotes ultrastructure were confirmed by TEM (Figure 4). Compared to untreated cells (Figure 4A), **Ox1** treatment induced significant morphological changes. At the IC_50_ concentration, there was a noticeable transition from the characteristic elongated morphology of the parasites to a more rounded shape (Figure 4B). Enhanced profiles of the endoplasmic reticulum can be also noticed, at lower concentrations of **Ox1** (Figure 4B). Regardless of the drug concentration, treated parasites exhibiting more than two flagella inside the flagellar pocket were commonly observed (Figure 4C). These changes are indicative of a compromised division process followed by loss of cell polarity and inner organization, as observed by SEM. Increased lipid droplets and swelling of parasite mitochondrion was observed in treated parasites (Figure 4D). Cells presenting different levels of damage, ranging from intact round-shaped cells (Figure 4D, 1) to ghost cell appearance, were observed in promastigotes treated with 2× IC_50_ **Ox1** (Figure 4D, 3). These data indicate that **Ox1** significantly affected the morphology and integrity of *L. infantum* promastigotes, ultimately leading the parasite to death.

### 2.6. Effects of ***Ox1*** on the Mitochondrial Membrane Potential of Promastigotes

**Figure 5:**
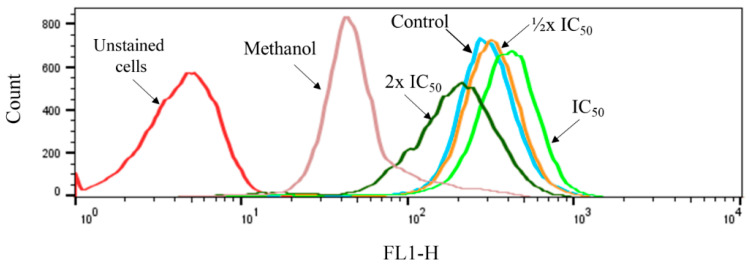
Representative histogram of untreated cells (control) and cells treated with half, once, and twice the IC_50_ of **Ox1**.

Because the mitochondrion of promastigotes was affected by the drug, we further investigated the effects of **Ox1** on the mitochondrial membrane potential by using Rhodamine 123 fluorescent probe (Figure 5). Compared to control cells, our results showed a discrete change in the membrane potential with values of variation index of +0.28 and −0.32 at IC_50_ and 2× IC_50_ of **Ox1**, respectively. No difference in the membrane potential was observed in cells treated with ½ IC_50_ of **Ox1**, with values of fluorescence intensity close to the control cells. Although the variation of fluorescence intensity of treated cells was almost similar to the control cells, it is important to keep in mind that organelles are very sensitive to minor variations in the concentration of ions and membrane potential, which can lead to organelle malfunction and cell death [55]. These results corroborate the swelling of mitochondria in promastigotes as observed by TEM (Figure 4B). It has been demonstrated that various molecules that act as sterol biosynthesis inhibitors affect the mitochondrion function [56,57,58]. De Macedo-Silva et al. [59] demonstrated that benzylamine derivatives, which disrupt the sterol biosynthesis pathway, induced ultrastructural alterations in treated *L. amazonensis* promastigotes similar to those found in our study. These alterations included loss of mitochondrial matrix content, vesiculation of the inner membrane, disorganization of the kinetoplast, and mitochondrial swelling [59]. Similarly, Paul et al. [58] showed that treatment with clotrimazole, an antifungal agent that also targets the ergosterol biosynthesis pathway, exhibited leishmanicidal effects against *L. donovani*, particularly showing high selectivity for intracellular amastigotes. According to the authors, clotrimazole compromises mitochondrial function, leading to membrane potential depolarization, reduced ATP levels, and interference with the cell cycle [58].

### 2.7. Effects of ***Ox1*** on the Ultrastructure of L. infantum Amastigotes

**Figure 6:**
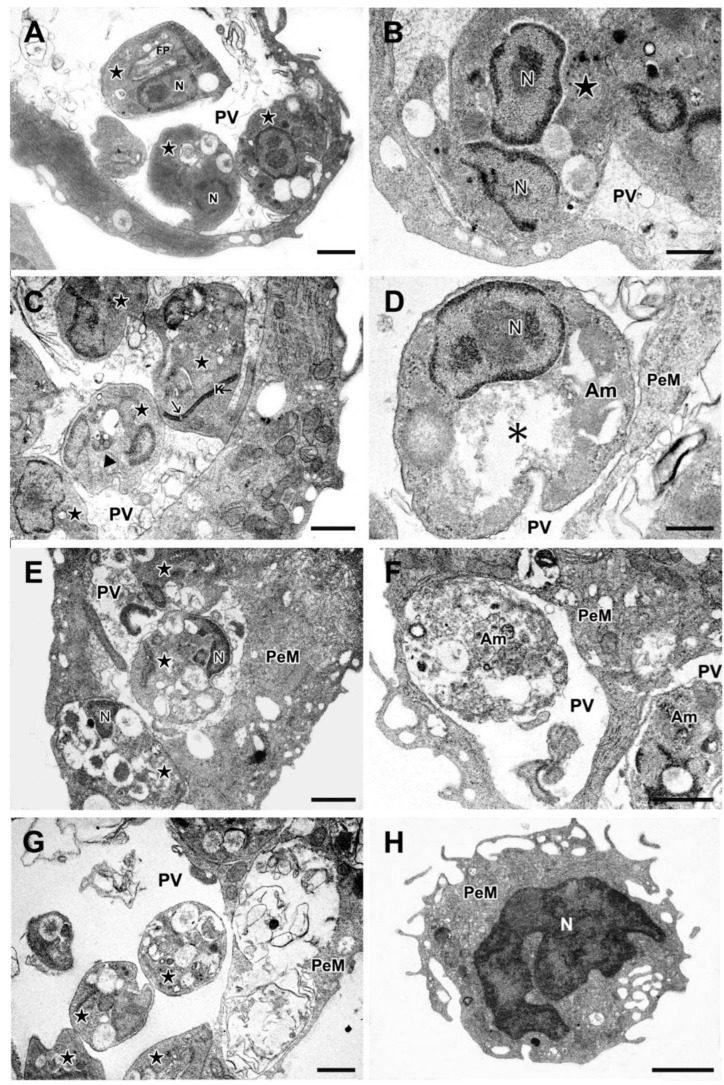
Transmission electron microscopy of the effects of **Ox1** on (PeM) infected with amastigote. (**A**,**B**) Untreated infected cells displaying several amastigotes (star) inside the parasitophorous vacuole (PV). (**B**) Detail of dividing amastigote (star) with preserved cellular structure. (**C**,**D**) Infected macrophages treated with IC_50_ **Ox1**. (**C**) Amastigotes (stars) presenting cytoplasmic vacuoles surrounded by membrane (arrowhead). Stretched kinetoplast showing partial rupture can be observed (arrow). (**D**) Amastigote with loss of cytoplasmic content, asterisk. (**E**,**F**) Infected PeM treated with 2× IC_50_ **Ox1**. (**E**) Amastigotes (star) presenting numerous membrane-bound vacuoles with or without internal content. Note the presence of high amounts of cellular debris inside the PV. (**F**) Detail of partially degraded amastigote (Am) inside the PV. (**G**) Infected macrophages treated with Amphotericin B. Amastigotes were indicated by stars. (**H**) Non-infected and non-treated macrophage. PV, parasitophorous vacuole; Am, amastigote; PeM, peritoneal macrophages infected with amastigote; FP, flagellar pocket; N, nucleus; K, kinetoplast. Bars: (**A**,**C**,**E**,**F**) =1µm; (**G**,**H**) =2 µm; (**B**,**D**) =500 nm.

In order to analyze the effects of **Ox1** on the ultrastructure of amastigote and host cells, TEM of PeM-infected cells was performed. Compared to untreated infected cells (Figure 6A,B), the treatment of infected PeMs induced drastic alterations in the morphology of intracellular amastigotes, even at low concentrations of **Ox1**. Electron-lucent areas (without a delineating membrane) (Figure 6D) and membrane-bounded vacuoles, with or without inner content (Figure 6C,E), were observed in the cytoplasm of amastigotes, especially at higher drug concentrations. The pronounced vacuolation of the cytoplasm and the appearance of multivesicular bodies are indicative of an ongoing autophagic process in amastigotes [60,61]. Autophagy serves as a crucial mechanism for cellular homeostasis and is one of the primary cellular responses to the stress induced by drugs in trypanosomatids [61,62,63]. In this regard, this mechanism might be triggered to cope with the damage induced by **Ox1** treatment; recycling damaged organelles [64]. However, exacerbated autophagy can evolve into cell death by necrosis [64,65]. The presence of parasitophorous vacuoles exhibiting a large amount of membrane debris and partially degraded material in treated cells (Figure 6C), compared to non-treated infected cells (Figure 6A), suggests ongoing parasite destruction. Indeed, at higher drug concentrations, completely damaged parasites were commonly observed (Figure 6F). Alterations in the kinetoplast, such as stretching and rupture of k-DNA, are also observed (Figure 6C). Overall, these effects on amastigotes are comparable to those observed in infected cells treated with Amphotericin B (Figure 6G). However, treatment with this reference drug induced pronounced damage to the host cells compared to PeMs treated with **Ox1** (Figure 6G). Moreover, comparing the infected and treated cells with uninfected and untreated macrophages (Figure 6H), it becomes evident that the observed alterations cannot solely be attributed to the drug treatment but are also influenced by the infection itself. This underscores the complexity of the cellular response to both infection and drug treatment [66].

### 2.8. Molecular Docking and Molecular Dynamics Calculations

In the process of identifying drug targets in parasites, it is imperative that they should be either absent in the host or substantially different from the host homolog counterpart in host cells and be crucial for parasite survival. It is also important to consider the stage of the pathogen’s life cycle in which the target is expressed [67]. In trypanosomatids, some vital metabolic pathways have been explored by many research groups searching for molecules that can interfere with key enzymes involved in the proliferation and survival of parasites [68]. In this work, we employed molecular docking and molecular dynamic simulations to gain insight into the potential mechanism of action and binding interactions of **Ox1** with some essential enzymes of *Leishmania* spp. In this regard, we selected some validated enzymatic targets involved in sterol biosynthesis (sterol 14-alpha demethylase, CYP51) [69], the de novo biosynthesis of pyrimidine nucleotides (dihydroorotate dehydrogenase, DHODH) [70], oxidative stress response (trypanothione reductase, TR) [71]; catabolism of amino acids (tyrosine aminotransferase, TAT) [72] and salvage pathway of pyrimidine (pteridine reductase, PTR1) [73]. These enzymes are crucial for parasite survival, and their deletion or inhibition usually leads the parasite to death [74].

**Figure 7:**
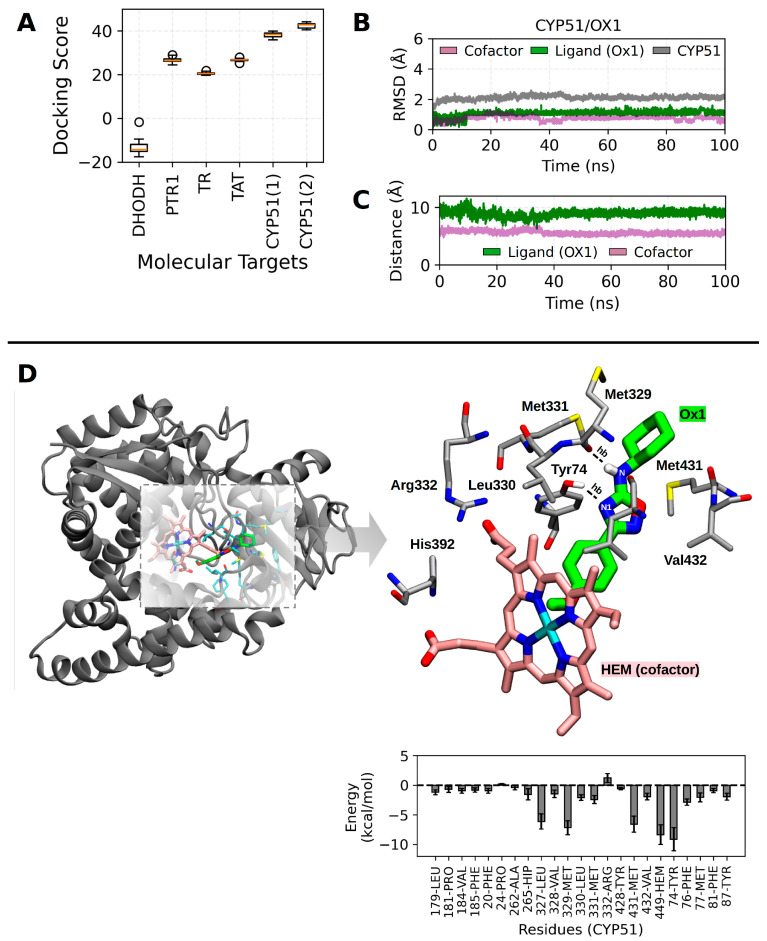
Summary of the in-silico results. (**A**) Docking scores obtained with GOLD software; label (1) corresponds to the scores obtained with the Chemscore scoring function, (2) corresponds to the modified version of this scoring function, with iron parameters. (**B**) RMSD profile during MD simulation. (**C**) Analysis of the center of mass distance during MD simulation. (**D**) The CYP51/**Ox1** complex is shown on the left and a detailed view of the binding site is shown on the right, showing the hydrophobic contacts and two hydrogen bonds (hb) between CYP51 and **Ox1** compound: (i) the hydroxyl group of Tyr74 and the nitrogen atom (N1) of **Ox1**, and (ii) the oxygen atom of Met329 and hydrogen atom attached to the nitrogen (N) to the **Ox1**. The interaction energy profile between CYP51 (per residue) and **Ox1** is shown below. It is important to note that the **Ox1** binding pose does not reflect the interaction analysis entirely since this analysis was carried out considering more than one binding pose. The structure of the CYP51 receptor in the cartoon model is shown in gray, and the carbon atoms of the binding site are in sticks model and colored in gray, whereas the carbon atoms of **Ox1** are shown in green. The carbon atoms of the heme propionate are shown in pink. The hydrogen atoms were omitted to improve visualization.

Our molecular docking screening with GOLD software (version 2023.2) showed that **Ox1** presents a high affinity for the CYP51 enzyme compared to other molecular targets analyzed (Figure 7A, and Table 3), with a docking score of 36.43 (and 39.77). Despite the high efficiency of this method in predicting bioactive binding poses, the predicted binding energy does not correlate well with experimental data [75]. Therefore, the ranking process requires a more rigorous filtering stage due to this inefficiency, which is why we perform post-docking stages. The first post-docking stage was MD simulations that aimed to address details about the dynamics and intermolecular interactions made by **Ox1** at the binding site of the selected molecular targets. In this sense, Guterres and co-workers showed that MD simulation is an essential post-docking step to discern active ligands from inactive ones [76]. Using the RMSD profile to evaluate the ligand-binding stability, they showed an improvement of 22% in ROC AUC, from an initial value of 0.68 (AutoDock Vina) to a final value of 0.83. Considering a time interval of 100 ns, the RMSD profile of **Ox1** at the CYP51 binding site showed that this compound did not undergo abrupt changes in its structure (Video S1). Similar behavior was also found for the CYP51 backbone and heme propionate (Figure 7B). In addition, the distance analysis showed that the **Ox1** remains close to its initial position, which means that strong, attractive interactions are present and restrict the movement of **Ox1** in the binding site of CYP51; a similar profile was found for the heme propionate group (Figure 7C). To understand the origin of these interactions (and, consequently, the structure–activity relationship), we performed a binding pose inspection of the last frame of MD simulation coupled to an interaction energy analysis (25 frames from the last 50 ns) and found that **Ox1** strongly interacts (interaction energy < −5 kcal/mol) with Tyr74, Leu327, Met329, Met431, and heme propionate (Figure 7D). Overall, this result suggests that hydrophobic contacts may be the origin of the conformational restraint and consequent stability of **Ox1** at the CYP51 binding site. Furthermore, such conformational restraint is reinforced by two hydrogen bonds between the Tyr74 and Met329 residues of CYP51 with **Ox1** (Figure 7D and Table S1). The NH group in **Ox1** donates a hydrogen bond to the oxygen atom in the Met329 backbone, while the N1 atom in **Ox1** accepts a hydrogen bond from the hydroxyl group in the Tyr74 side chain. In addition, the methoxy (OCH_3_) moiety is in very close contact with the cofactor, suggesting a strong hydrophobic contact. These findings reinforce the hypothesis presented in the last paragraph of Section 2.3.

MD simulations for the PTR1/**Ox1**, TAT/**Ox1**, and TR/**Ox1** complexes were carried out for comparison, and all results are available in the supporting information. Despite the high stability of **Ox1** at the binding sites of PTR1 and TR (Figures S4 and S5), the calculated per residue interaction energy along the MD simulation was lower than those obtained by CYP51 (Figure 7D and Figure S6). Regarding the TAT/**Ox1** complex, the **Ox1** compound showed a high level of instability (Figures S4 and S5) and a low occurrence of hydrogen bonds (Table S1), as well as a low few attractive interactions at the binding site when compared to CYP51 (Figure S6). The second post-docking step was to calculate the binding enthalpy (ΔH_bind_) in the light of quantum mechanics theory, considering 25 frames from the last 50 ns of the MD simulation. In this sense, the strength of interactions in protein–ligand complexes is impacted by both entropic and enthalpic terms. However, the specificity of ligands to their respective binding pockets is mainly determined by enthalpy. Therefore, ligands designed for a particular receptor tend to have a beneficial binding enthalpy due to specific intermolecular interactions [77,78]. In the present study, the calculations of binding enthalpy showed CYP51 as the high-affinity target with which **Ox1** interacts, with ΔH_bind_ = −50.060 kcal/mol (Table 3). The MM/GBSA calculations corroborate the CYP51 as the high-affinity target, with ΔG_bind_ = −42.686 kcal/mol (Table 3).

The molecular docking and dynamic analysis of **Ox1** support our biological and ultrastructural data, showing that this compound may act as an inhibitor of CYP51, significantly affecting the biosynthesis of ergosterol in this parasite and causing the ultrastructural changes observed by SEM and TEM. CYP51 is an enzyme belonging to the highly conserved cytochrome P450 family, catalyzing the oxidative removal of the 14*α*-methyl group from one or more of the five naturally occurring cyclized sterol precursors [79]. This enzyme not only prevents the toxic accumulation of methylated sterol precursors but also participates in the synthesis of essential membrane components and regulatory molecules in fungi and protozoans [80]. Although DNDi has discouraged the use of compounds targeting sterol 14-alpha demethylase in trypanosomatids due to clinical trial failures [81], previous studies on VN1 and VFV, two oxadiazole-containing compounds that have proven to be strong inhibitors of CYP51, have demonstrated efficacy in curing Chagas disease in murine models infected with *T. cruzi* [81,82,83].

## 3. Materials and Methods

### 3.1. Synthesis

#### 3.1.1. Synthesis of 3-Aryl-5-cyclohexylamino-1,2,4-oxadiazole Derivatives (**Ox**, **Ox1**, **Ox2**, **Ox3**, **Ox4**) [28]

A mixture of 1 mmol of arylamidoximes and dicyclohexylcarbodiimide (1.5 mmol) in DMF (0.5 mL) was irradiated in a closed vessel at 120 °C and 150 W for 10 min. After cooling, the residue was evaporated under reduced pressure, and the crude material was purified by column chromatography on silica gel (hexane:EtOAc, 8:2) to give the products **Ox**–**Ox4** (Scheme 1).

*N-Cyclohexyl-3-(phenyl)-1,2,4-oxadiazol-5-amine* (**Ox**): Yield = 85%; colorless solid; Mp 125–127 °C [Lit. 124–126 °C] [28]; R_f_ = 0.3 (hexane-EtOAc, 8:2). The NMR spectroscopy was recorded and compared to previously reported data [28].

*N-Cyclohexyl-3-(4-methoxyphenyl)-1,2,4-oxadiazol-5-amine* (**Ox1**): Yield = 76%; yellow solid; Mp 109–110 °C {Lit. 107–109 °C} [28]; R_f_ = 0.3 (hexane-CH_2_Cl_2_, 1:1). The NMR spectroscopy was recorded and compared to previously reported data [28].

*N-Cyclohexyl-3-(3-nitrophenyl)-1,2,4-oxadiazol-5-amine* (**Ox2**): Yield = 84%; yellow solid; Mp 131–133 °C {Lit. 130–131 °C} [84]; R_f_ = 0.3 (hexane-CH_2_Cl_2_, 1:1). The NMR spectroscopy was recorded and compared to previously reported data [84].

*N-Cyclohexyl-3-(4-nitrophenyl)-1,2,4-oxadiazol-5-amine* (**Ox3**): Yield = 81%; yellow solid; Mp 201–203 °C {Lit. 201–202 °C} [84]; R_f_ = 0.3 (hexane-CH_2_Cl_2_, 1:1). The NMR spectroscopy was recorded and compared to previously reported data [84].

*N-Cyclohexyl-3-(4-methylphenyl)-1,2,4-oxadiazol-5-amine* (**Ox4**): Yield = 79%; colorless solid; Mp 144–146 °C {Lit. 142–144 °C} [28]; R_f_ = 0.4 (hexane-CH_2_Cl_2_, 1:1). The NMR spectroscopy was recorded and compared to previously reported data [28].

#### 3.1.2. Synthesis of the *N*-Propargyl-1,2,4-oxadiazole Derivatives (**Ox5**, **Ox6**, **Ox7**) [20]

Amine-cyclohexyl-1,2,4-oxadiazoles (1 mmol) were dissolved in dry DMF (2 mL), followed by addition of potassium *tert*-butoxide (2 equiv). Then, propargyl bromide (4 equiv) was added, and the mixture was stirred at room temperature for 2 h. The reaction mixture was filtered and washed with dichloromethane/H_2_O, and the solvents were evaporated under reduced pressure. The products were purified by column chromatography using hexane:dichloromethane (2:8) to give the products **Ox5**–**Ox7** (Scheme 1). The chemical structures of 1,2,4-oxadiazoles (**Ox5**–**Ox7**) were confirmed by ^1^H and ^13^C nuclear magnetic resonance [20].

*N-Cyclohexyl-3-phenyl-N-(prop-2-yn-1-yl)-1,2,4-oxadiazol-5-amine* (**Ox5**): Yield = 92%; yellow solid; Mp 112–114 °C {Lit. 112–115 °C} [20]; R_f_ = 0.4 (hexane-EtOAc, 8:2).

*N-Cyclohexyl-3-(4-nitrophenyl)-N-(prop-2-yn-1-yl)-1,2,4-oxadiazol-5-amine* (**Ox6**): Yield = 90%; yellow solid; Mp 108–110 °C {Lit. 104–105 °C} [20]; R_f_ = 0.3 (hexane-EtOAc, 8:2).

*N-Cyclohexyl-3-(3-nitrophenyl)-N-(prop-2-yn-1-yl)-1,2,4-oxadiazol-5-amine* (**Ox7**): Yield = 93%; yellow solid; Mp 94–96 °C {Lit. viscous oil} [20]; R_f_ = 0.3 (hexane-EtOAc, 8:2).

### 3.2. Physiochemical Assay

The prediction of the physicochemical properties of compounds **Ox1**–**Ox7** was conducted using the SwissADME platform (http://www.swissadme.ch, accessed on 30 March 30 2022). Amongst various physicochemical properties, this tool facilitates the assessment of drug-likeness for a compound, following the Lipinski’s rule of five [36]. The rules established by Veber et al. [37] for oral bioavailability were also considered in the analyses, encompassing the number of rotatable bonds (n-ROTB ≤ 10) and topological polar surface area (TPSA ≤ 140 A^2^).

### 3.3. Cytotoxicity Assay in Mammalian Cells

The stock solutions of the compounds (**Ox1**–**Ox7**) were prepared at a concentration of 50 mg/mL in 100% dimethylsulfoxide (DMSO) and subsequently diluted in culture medium to ensure that the DMSO concentration did not exceed 1%, which is recognized as non-cytotoxic to mammalian cells. The cytotoxic potential of **Ox1**–**Ox7** was first evaluated in fibroblasts (L929) and macrophages (J774.G8). For this, 5 × 10^5^ cells/mL were seeded in 96-well microplates containing 100 μL of DMEM or RPMI-1640 medium supplemented with 10% fetal bovine serum (FBS) and cultivated for 24 h at 37 °C, under 5% CO_2_ atmosphere. Subsequently, different concentrations of the tested compounds (6.25–200 µM) were added to the adhered cells. Untreated cells were considered as negative control. The cell viability was measured by spectrometry after 48 h of cultivation using MTT (Sigma-Aldrich, St. Louis, MO, USA) methodology described by Mosmann [85]. The optical densities (OD) of the samples were read in a Glomax Multiplate reader (Promega, Madison, WI, USA) at 540 nm. Each assay was performed in triplicate in three independent experiments. The cytotoxic concentration of the compounds for 50% of the cells (CC_50_) was calculated by regression analysis using IBM SPSS Statistics 20 software. The selectivity index (SI) for each compound was calculated as the ratio between the CC_50_ values obtained for J774.G8 macrophages and fibroblast and the IC_50_ values for promastigotes. The compound with a higher value of SI was selected for cytotoxicity evaluation in macrophages obtained from BALB/c mice peritoneal exudate (PeM), following the methodology described above.

### 3.4. Promastigotes Culture

Promastigote forms of *L. infantum* (BH46 strain) were cultivated at 27 °C in Schneider’s medium, supplemented with 10% (*v*/*v*) heat-inactivated fetal bovine serum (FBS, Gibco, Waltham, MA, USA), 100 U/mL penicillin, and 0.01 mg/mL streptomycin. Parasites in the log phase were used in the test of drugs against promastigotes, whereas those obtained in the stationary phase were used to infect peritoneal macrophages.

### 3.5. In Vitro Assay of the Effects of ***Ox1**–**Ox7*** on L. infantum Promastigotes

*L. infantum* promastigotes at density of 1 × 10^6^ cells/mL were dispensed into 96-well optical bottom white plates (Thermo Scientific, Waltham, MA, USA) and incubated in the absence or presence of various concentrations of **Ox1**–**Ox7** (6.25–200 µM) diluted in Schneider’s medium supplemented with 10% FBS, for 24 and 48 h, at 27 °C. The viability of promastigotes was assessed through ATP production using the CellTiter-Glo^®^ Luminescent Cell Viability assay (Promega, Madison, WI, USA), in accordance with the manufacturer’s instructions. The 50% inhibitory concentration (IC_50_) value was determined after 48 h of drug treatment through nonlinear analysis using IBM SPSS Statistics 20 software.

### 3.6. The Effects of 1,2,4-Oxadiazole Selected Derivative on Amastigote Forms

The most promissory compound (with higher SI) was chosen to evaluate its effects on the peritoneal macrophage (PeM) infection by *L. infantum*. For this, PeMs were seeded at 10^6^ cell/mL in 24-well culture plates containing RPMI supplemented with 10% FBS at 37 °C and 5% de CO_2_ atmosphere. After initial cultivation, non-adherent cells were removed by washing with fresh RPMI medium. Subsequently, PeMs were infected with stationary promastigote forms of *L. infantum* at 1:20 PeM/promastigotes ratio. The cells were cultivated for 14 h at 37 °C, and then the non-internalized parasites were removed by washing. The parasites were treated or not, for 24 h, with the selected compound at concentrations of 8.24, 16.49, and 32.98 µM, which correspond to ¼ IC_50_, ½ IC_50,_ and IC_50_ for promastigote, respectively. Untreated–infected cells and cells treated with the reference drugs antimoniate of meglumine (Glu, IC_50_ = 346.19 µM) and Amphotericin B (AmB, IC_50_ = 10.55 µM) were used as negative and positive controls, respectively [86,87]. After incubation time, the samples were washed in PBS, fixed with methanol, and stained with Giemsa. The percentage of infected macrophages was determined by counting 150 randomly chosen cells in duplicate, and the survival index (SuI) was calculated by multiplying the percentage of infected cells by the mean of amastigotes in infected cells. The IC_50_ concentration for amastigotes was calculated from the total number of amastigotes in the 300 counted cells, as described above, and the SI of most promising compound for promastigotes and amastigotes was calculated as a ratio of CC_50_ for PeM/IC_50_ of amastigote forms. The tests were carried out in duplicate in three independent experiments.

### 3.7. Ultrastructural Assay

To identify alterations in the morphology of promastigotes and amastigotes, scanning (SEM) and transmission (TEM) electron microscopy assays were performed. For SEM, *L. infantum* promastigotes were treated with once or twice the IC_50_ concentration of **Ox1** for 48 h. Subsequently, the parasites were harvested by centrifugation, washed with 100 mM PBS, pH 7.2, fixed at room temperature, for 1 h in 2.5% glutaraldehyde solution diluted in 0.1 M cacodylate buffer, pH 7.2. Cells were adhered to coverslips pre-coated with poly-L-lysine, post-fixed for 1 h with a solution containing 1% osmium tetroxide, 0.8% potassium ferricyanide, and 5 mM calcium chloride diluted in 0.1 M cacodylate buffer, pH 7.2, in light-protected environment. Then, the cells were dehydrated in graded ethanol series (15–100%) for 10 min at room temperature. Samples were critical-point dried in a LEICA EM CPD300 apparatus (Leica Microsystems, Wetzlar, Hessen, DE, Germany) and then coated with 20 nm gold and observed in a JEOL T-200 scanning electron microscope (JEOL Ltd., Tokyo, Japan). For MET, promastigotes and infected PeMs were treated or not with once or twice IC_50_ concentration of **Ox1**, fixed and post-fixed, as described above. For infected–adhered peritoneal macrophages, after the post-fixation, the cells were scraped off the culture dishes with a rubber policeman. Promastigotes and infected cells were harvested by centrifugation and dehydrated in a graded acetone series. After dehydration in acetone, the samples were embedded and polymerized in Epon resin. Ultrathin sections (60–70 nm) were obtained, stained with uranyl acetate and lead citrate, and observed in a FEI Tecnai Spirit Biotwin G2 Transmission electron microscope (Thermo Fisher Scientific, Waltham, MA, USA) at 80 KV.

### 3.8. Effects of ***Ox1*** on the Parasite Mitochondria

To evaluate the effects of **Ox1** on the mitochondrial membrane potential (Δψm) of the parasite, *L. infantum*, promastigotes (1 × 10^6^ cells/mL) were treated or not (negative control) with ½ IC_50_, IC_50_, and 2× IC_50_ concentrations of **Ox1** for 48 h at 27 ºC. Afterward, the cells were centrifuged and stained with Rhodamine 123 (10 µg/mL, molecular probes, Invitrogen™, Waltham, MA, USA) in PBS for 15 min at 27 °C, in the dark [88]. The fluorescence intensity of Rhodamine 123 stained cells was analyzed using a BD FACSCalibur cytometer with an FL1-H detector (Becton, Dickinson and Company, Franklin Lakes, NJ, USA). The changes in the fluorescence intensity for fluorochrome were determined by the variation index calculated as follows: (MT − MC)/MC where MT is the mean fluorescence intensity of treated cells and MC is the mean of fluorescence of control cells without treatment. A total of 20,000 events were acquired, and the data were analyzed with FLOWJO software version 10.2.

### 3.9. Molecular Docking

The molecular docking approach was performed to predict the relative binding affinity of the **Ox1** compound against the following molecular targets of *L. infantum*: (i) sterol 14-alpha demethylase (PDB ID 3L4D [89]); (ii) dihydroorotate reductase; (iii) tyrosine aminotransferase (PDB ID 4IX8 [90]); (iv) pteridine reductase; and (v) trypanothione reductase (PDB ID 2YAU [91]). There is no structural information about dihydroorotate reductase and pteridine reductase from *L. infantum* on Protein Data Bank, so we carried out a homology modeling protocol to obtain them (see the supporting information for more details). These geometries (and the resulting ones from the homology modeling protocol) were pre-processed considering the following steps: (i) waters and ions were removed while co-factors were maintained; (ii) missing hydrogens and the protonated state of titratable residues were added, and predicted, respectively, by using the H++ web service [92]; (iii) the resulting geometries were relaxed over 20,000 of conjugate gradient steps in the NAMD software (version 2.13) [93] (see the supporting information for more details). The 3D structure of the **Ox1** compound was generated using the MarvinSkecth software (version 19.21.7), and the molecular docking calculations were performed using the GOLD software [94], configured according to the following parameters: (i) the binding sites were delimited using the structural information of co-crystallized ligands/substrates; we consider residues close to the center of mass of the co-crystallized ligands as active; (ii) Chemscore was chosen as the scoring function, configured for exhaustive search; a modified version of this scoring function that includes extra parameters (iron parameters) for the CYP51 receptor was also used to evaluate the interaction of the **Ox1** compound at the CYP51 binding site. The best-ranked protein–ligand complex was simulated by molecular dynamics (MD) in the NAMD software [93].

### 3.10. MD Simulations and MD Trajectory Analysis

The protein structures were parameterized according to the ff19SB force field [95], while the **Ox1** compound and cofactors were parameterized with the GAFF2 force field [96] and net charges according to AM1BCC method. The structure of CYP51 contains a heme group in the binding site, so we used the AMBER parameters database maintained by Richard Bryce at the University of Manchester to parameterize this molecule. The resulting geometry was then inserted into a box of water containing TIP3 water molecules, and counter-ions were added to achieve neutrality. Furthermore, the MD simulations were configured as follows: (i) periodic boundary conditions; (ii) restriction of vibration for covalent bonds involving hydrogen atoms, HOH angles, and the OH bond distance of TIP3P water molecules; (iii) time step equal to 2 fs; (iv) electrostatic interaction cutoff of 12 Å for all steps of the simulation; and (v) Particle Mesh Ewald method was used for long-range electrostatic interaction. The starting geometries were submitted to minimization, heating from 0 to 310 K, and pressurization steps as follows: (i) the systems were relaxed over 20,000 conjugate gradient steps, in which the first 5000 steps were performed for non-protein atoms (i.e., ions, water molecules, ligand, and cofactors) and remaining 15,000 steps were carried out for all atoms; (ii) the systems were heated to 100 K in 40 ps considering harmonic restraints with a force constant of 30 kcal/mol on Cα atoms and 10 kcal/mol on C and N atoms of the protein backbones; (iii) the systems were simulated for 100 ps with a force constant of 15 kcal/mol on Cα atoms and 5 kcal/mol on C and N atoms of the protein backbones for 100 ps; (iv) the temperatures of the systems were increased to the desired value of 310 K after simulating for 200 ps by keeping weak harmonic restraints with a force constant of 7.5 kcal/mol on Cα atoms and of 2.5 kcal/mol on C and N atoms of the protein backbones. The thermalization phase (NVT ensemble) was followed by a pressurization phase (NPT ensemble). The systems were then simulated for 100 ps; there were weak harmonic restraints with a force constant of 3.75 kcal/mol on Cα atoms and 1.25 kcal/mol on C and N atoms. Then, a stage of 200 ps was carried out, and only Cα atoms of the proteins were restrained with a force constant of ~1.5 kcal/mol. A pressure of 1 atm was maintained using the Langevin piston barostat, and the systems were simulated for 100 ns without restraints, and frames were captured every 10 ps. The trajectory analysis of MD simulations was carried out using the CPPTRAJ [97] (version 6.18.1) software. In addition, we re-scored the free energies of binding of the simulated complexes in the light of the MM/GBSA method; at last, we also evaluated the enthalpies of binding for these complexes [93,95,96,97,98] (see the Supporting Information for more details).

### 3.11. Statistical Assays

Probit regression analyses were performed using the IBM SPSS Statistic 25 software program in order to determine the values of CC_50_ and IC_50_. A one-way analysis of variance (ANOVA) was performed to test for statistical significance (GraphPad Prism). All tests were performed using the GraphPad Prism 5 program (GraphPad Software Inc. 2007, San Diego, CA, USA), and the *p*-value < 0.05 was considered significant.

### 3.12. Ethical Considerations

Male BALB/c mice were obtained from the animal facility of the Aggeu Magalhães Institute located in the city of Recife, Pernambuco, Brazil. This research was conducted in accordance with the ethical principles adopted by Brazilian legislation 11.794/2008 regarding the use of experimental animals and approved by the Aggeu Magalhães Institute/Oswaldo Cruz Foundation Animal Research Ethics Committee (No. 146/2019).

## 4. Conclusions

The compounds **Ox1**–**Ox7** exhibited good physicochemical properties useful for the development of oral formulations. All derivatives showed the ability to inhibit the viability of *L. infantum* promastigotes, with CC_50_ values ≥ 160 µM for mammalian cells. Among the tested compounds, **Ox1** was the most selective against promastigotes with low cytotoxicity to mammalian cells. This compound was also highly effective and selective against amastigotes (SI = 61.7). Our ultrastructural analysis demonstrated that **Ox1** impairs cell division and causes damage to promastigotes mitochondrion, whereas, in amastigotes, morphological changes are indicative of an autophagic process at low concentrations of the drug, probably to overcome its deleterious action. However, at high concentrations of **Ox1**, amastigotes evolve to cell death. The morphological changes observed in promastigotes and amastigotes suggest that the ergosterol synthesis may be inhibited by drug treatment. Consistently, our docking molecular and molecular dynamic simulations showed that **Ox1** strongly binds CYP51 and maintains its structural stability during its interaction with the enzyme binding site. Taken together, these results suggest that **Ox1** has great potential as a candidate for chemotherapy against *L. infantum*, combining physicochemical features with its high selectivity against amastigotes and low cytotoxicity towards mammalian cells.

## Figures and Tables

**Table 1:** Predicted physicochemical properties of **Ox1**–**Ox7** and miltefosine.

Compounds	Lipinski Rules	MW (≤500) g/mol	HBA (≤10)	HBD (≤5)	LogP (≤5)	n-ROTB (≤10)	TPSA (≤140 A^2^)
**Ox1**	Yes	273.33	4	1	3.10	4	60.18
**Ox2**	Yes	288.30	5	1	2.64	4	96.77
**Ox3**	Yes	288.30	5	1	2.73	4	96.77
**Ox4**	Yes	257.33	3	1	3.32	3	50.95
**Ox5**	Yes	267.33	3	0	3.57	3	42.16
**Ox6**	Yes	326.35	5	0	3.26	5	87.98
**Ox7**	Yes	326.35	5	0	3.28	5	87.98
Miltefosine	Yes	407.57	4	0	3.35	20	68.40

MW—molecular weight; HBA—number of hydrogen bond acceptors; HBD—number of hydrogen bond donors; LogP—octanol–water partition coefficient (lipophilicity); n-ROTB—number of rotatable bonds and TPSA—topological polar surface area.

**Table 2:** Effect of 1,2,4-oxadiazole derivatives on mammalian cells and promastigotes of *L. infantum*.

Compound	Mammalian Cell Type		Pro	SI	
	CC_50_ (µM)		IC_50_ (µM)	Pro/L929	Pro/J774.G8
	L929	J774.G8			
**Ox1**	320 ± 4.7	293.1 ± 5.5	32.9 ± 2.2	9.7	8.9
**Ox2**	364 ± 6.1	285.4 ± 7.0	220 ± 3.6	1.7	1.2
**Ox3**	356 ± 6.2	>200	336 ± 12.1	1.1	n.d.
**Ox4**	290 ± 10.0	>200	174.4 ± 12.3	1.7	n.d.
**Ox5**	375 ± 15.3	160 ± 6.5	84.7 ± 3.6	4.4	1.8
**Ox6**	338 ± 31.1	231 ± 8.1	92.2 ± 1.9	3.7	2.5
**Ox7**	>200	197 ± 6.0	98.2 ± 1.4	n.d.	2.0

CC_50_—50% cytotoxic concentration for mammalian cells (48 h of treatment); IC_50_—50% inhibitory concentration for promastigotes (after 48 of treatment); SI—selectivity index calculated by the ratio CC_50_/IC_50_; Pro—promastigotes; n.d.—non-determined. The values represent mean ± standard deviation of three independent experiments in triplicate.

**Table 3:** Summary of relative binding affinity calculations. The data shown in the second column is in the GOLD scoring unit and corresponds to the highest-ranked docking pose, while the data in the third and fourth columns are in kcal/mol, followed by the mean error. The ΔG_bind_ and ΔH_bind_ were calculated considering the same 25 frames from the last 50 ns of MD simulation.

Receptor	Docking Score	ΔG_bind_ (MM/GBSA)	ΔH_bind_ (PM7)
CYP51	36.43 ^(a)^, 39.77 ^(b)^ (1)	−42.686 ± 0.636 (1)	−50.060 ± 0.875 (1)
TAT	27.30 (2)	−14.663 ± 0.593 (4)	−18.702 ± 0.885 (4)
PTR1	26.17 (3)	−37.946 ± 0.822 (2)	−40.471 ± 1.076 (2)
TR	20.40 (4)	−25.632 ± 0.811 (3)	−25.588 ± 0.765 (3)
DHODH	−9.59 (5)	-	-

^(a)^ Chemscore configured with default parameters; ^(b)^ Chemscore with iron parameters. (1)–(5) The numbering between the brackets refers to the ranking of the poses.

## Data Availability

The data are contained within the article and Supplementary Materials.

## Supplementary Materials

The following supporting information can be downloaded at: https://www.mdpi.com/article/10.3390/molecules29194654/s1, Figure S1: Effects of **Ox2**–**Ox7** on the viability of Fibroblasts (L929) after 48 h of treatment; Figure S2: Effects of **Ox2**–**Ox7** on the viability of macrophages (J774.G8) after 48 h of treatment; Figure S3: Effects of **Ox2**–**Ox7** on *L. infantum* promastigotes after 24 and 48 h of treatment; Homology Modeling; Figure S4: RMSD profile of the MD trajectories of the complexes TAT/**Ox1** (A), PTR1/**Ox1** (B), and TR/**Ox1** (C); Figure S5. Center of mass distance analysis of the MD trajectories of the complexes TAT/**Ox1** (A), PTR1/**Ox1** (B), and TR/**Ox1** (C); Figure S6: Interaction energy profile of the MD trajectories of the complexes TAT/**Ox1** (A), PTR1/**Ox1** (B), and TR/**Ox1** (C); Table S1. Hydrogen bonds profile between the **Ox1** compound and molecular targets of *L. infantum* during MD simulation; Video S1: RMSD Profile of **Ox1** at the CYP51 Binding Site Demonstrates Structural Stability Over 100 ns Interval. Refs. [93,95,96,97,98] are cited in Supplementary Materials.

## References

[1] Wamai R.G., Kahn J., McGloin J., Ziaggi G. (2020). Visceral Leishmaniasis: A Global Overview. J. Glob. Health Sci..

[2] Van Griensven J., Diro E. (2012). Visceral Leishmaniasis. Infect. Dis. Clin. N. Am..

[3] Soni V., Chandel S., Pandey V., Asati S., Jain P., Jain A., Tekade R.K., Tekade R.K. (2019). Chapter 8—Novel Therapeutic Approaches for the Treatment of Leishmaniasis. Biomaterials and Bionanotechnology.

[4] Mann S., Frasca K., Scherrer S., Henao-Martínez A.F., Newman S., Ramanan P., Suarez J.A. (2021). A Review of Leishmaniasis: Current Knowledge and Future Directions. Curr. Trop. Med. Rep..

[5] WHO Leishmaniasis. https://www.who.int/news-room/fact-sheets/detail/leishmaniasis.

[6] Yasmin H., Adhikary A., Al-Ahdal M.N., Roy S., Kishore U. (2022). Host–Pathogen Interaction in Leishmaniasis: Immune Response and Vaccination Strategies. Immuno.

[7] Moore E.M., Lockwood D.N. (2010). Treatment of Visceral Leishmaniasis. J. Glob. Infect. Dis..

[8] Field M.C., Horn D., Fairlamb A.H., Ferguson M.A.J., Gray D.W., Read K.D., De Rycker M., Torrie L.S., Wyatt P.G., Wyllie S. (2017). Anti-Trypanosomatid Drug Discovery: An Ongoing Challenge and a Continuing Need. Nat. Rev. Microbiol..

[9] Santos S.S., de Araújo R.V., Giarolla J., Seoud O.E., Ferreira E.I. (2020). Searching for Drugs for Chagas Disease, Leishmaniasis and Schistosomiasis: A Review. Int. J. Antimicrob. Agents.

[10] Kapil S., Singh P.K., Silakari O. (2018). An Update on Small Molecule Strategies Targeting Leishmaniasis. Eur. J. Med. Chem..

[11] Dhameliya T.M., Chudasma S.J., Patel T.M., Dave B.P. (2022). A Review on Synthetic Account of 1,2,4-Oxadiazoles as Anti-Infective Agents. Mol. Divers..

[12] Shukla P., Verma A., Mishra P. (2017). Significance of Nitrogen Heterocyclic Nuclei in the Search of Pharmacological Active Compounds. New Perspective in Agricultural and Human Health.

[13] Nagaraja O., Bodke Y.D., Pushpavathi I., Ravi Kumar S. (2020). Synthesis, Characterization and Biological Investigations of Potentially Bioactive Heterocyclic Compounds Containing 4-Hydroxy Coumarin. Heliyon.

[14] Barbuceanu S.-F., Rosca E.-V., Apostol T.-V., Socea L.-I., Draghici C., Farcasanu I.C., Ruta L.L., Nitulescu G.M., Iscrulescu L., Pahontu E.-M. (2023). New Heterocyclic Compounds from Oxazol-5(4H)-One and 1,2,4-Triazin-6(5H)-One Classes: Synthesis, Characterization and Toxicity Evaluation. Molecules.

[15] Biological Activity of Oxadiazole and Thiadiazole Derivatives|Applied Microbiology and Biotechnology. https://link.springer.com/article/10.1007/s00253-022-11969-0.

[16] Biernacki K., Daśko M., Ciupak O., Kubiński K., Rachon J., Demkowicz S. (2020). Novel 1,2,4-Oxadiazole Derivatives in Drug Discovery. Pharmaceuticals.

[17] Carbone M., Li Y., Irace C., Mollo E., Castelluccio F., Di Pascale A., Cimino G., Santamaria R., Guo Y.-W., Gavagnin M. (2011). Structure and Cytotoxicity of Phidianidines A and B: First Finding of 1,2,4-Oxadiazole System in a Marine Natural Product. Org. Lett..

[18] Zhou M., Boulos J.C., Omer E.A., Klauck S.M., Efferth T. (2023). Modes of Action of a Novel C-MYC Inhibiting 1,2,4-Oxadiazole Derivative in Leukemia and Breast Cancer Cells. Molecules.

[19] Atmaram Upare A., Gadekar P.K., Sivaramakrishnan H., Naik N., Khedkar V.M., Sarkar D., Choudhari A., Mohana Roopan S. (2019). Design, Synthesis and Biological Evaluation of (E)-5-Styryl-1,2,4-Oxadiazoles as Anti-Tubercular Agents. Bioorgan. Chem..

[20] de Oliveira V.N.M., do Amaral Moura C.F., dos Santos Peixoto A., Ferreira V.P.G., Araújo H.M., Pimentel L.M.L.M., do Ó Pessoa C., Nicolete R., Dos Anjos J.V., Sharma P.P. (2021). Synthesis of Alkynylated 1,2,4-Oxadiazole/1,2,3-1*H*-Triazole Glycoconjugates: Discovering New Compounds for Use in Chemotherapy against Lung Carcinoma and *Mycobacterium tuberculosis*. Eur. J. Med. Chem..

[21] Mohamed M.F.A., Marzouk A.A., Nafady A., El-Gamal D.A., Allam R.M., Abuo-Rahma G.E.-D.A., El Subbagh H.I., Moustafa A.H. (2020). Design, Synthesis and Molecular Modeling of Novel Aryl Carboximidamides and 3-Aryl-1,2,4-Oxadiazoles Derived from Indomethacin as Potent Anti-Inflammatory iNOS/PGE2 Inhibitors. Bioorgan. Chem..

[22] Parikh P.H., Timaniya J.B., Patel M.J., Patel K.P. (2020). Design, Synthesis, and Characterization of Novel Substituted 1,2,4-Oxadiazole and Their Biological Broadcast. Med. Chem. Res..

[23] Cottrell D.M., Capers J., Salem M.M., DeLuca-Fradley K., Croft S.L., Werbovetz K.A. (2004). Antikinetoplastid Activity of 3-Aryl-5-Thiocyanatomethyl-1,2,4-Oxadiazoles. Bioorgan. Med. Chem..

[24] Fernandes F.S., Santos H., Lima S.R., Conti C., Rodrigues M.T., Zeoly L.A., Ferreira L.L.G., Krogh R., Andricopulo A.D., Coelho F. (2020). Discovery of Highly Potent and Selective Antiparasitic New Oxadiazole and Hydroxy-Oxindole Small Molecule Hybrids. Eur. J. Med. Chem..

[25] Dai H., Chen J., Li G., Ge S., Shi Y., Fang Y., Ling Y. (2017). Design, Synthesis, and Bioactivities of Novel Oxadiazole-Substituted Pyrazole Oximes. Bioorgan. Med. Chem. Lett..

[26] Meher D.K., Nayak A.K., Singh A.K., Pathak M. (2020). Synthesis, Evaluation & Pharmacological Action of Oxadiazole Derivatives. J. Pharmacogn. Phytochem..

[27] Pinheiro C.V.G., da Silva W.M.B., Rodrigues J.P.V., Rocha Y.M., Teixeira M.J., de Oliveira R.N., de Souza N.V., Nicolete R. (2022). Anti-*Leishmania infantum* in Vitro Effect of n-Cyclohexyl-1,2,4-Oxadiazole and Its ADME/TOX Parameters. J. Parasit. Dis..

[28] Oliveira V.N.M., dos Santos F.G., Ferreira V.P.G., Araújo H.M., do Ó Pessoa C., Nicolete R., de Oliveira R.N. (2018). Focused Microwave Irradiation-Assisted Synthesis of N-Cyclohexyl-1,2,4-Oxadiazole Derivatives with Antitumor Activity. Synth. Commun..

[29] Pace A., Buscemi S., Piccionello A.P., Pibiri I., Scriven E.F.V., Ramsden C.A. (2015). Recent Advances in the Chemistry of 1,2,4-Oxadiazolesa. Advances in Heterocyclic Chemistry.

[30] Mezeiova E., Janockova J., Andrys R., Soukup O., Kobrlova T., Muckova L., Pejchal J., Simunkova M., Handl J., Micankova P. (2021). 2-Propargylamino-Naphthoquinone Derivatives as Multipotent Agents for the Treatment of Alzheimer’s Disease. Eur. J. Med. Chem..

[31] Lutsenko K., Hagenow S., Affini A., Reiner D., Stark H. (2019). Rasagiline Derivatives Combined with Histamine H3 Receptor Properties. Bioorgan. Med. Chem. Lett..

[32] Santoso K.T., Menorca A., Cheung C.-Y., Cook G.M., Stocker B.L., Timmer M.S.M. (2019). The Synthesis and Evaluation of Quinolinequinones as Anti-Mycobacterial Agents. Bioorgan. Med. Chem..

[33] Reguera R.M., Pérez-Pertejo Y., Gutiérrez-Corbo C., Domínguez-Asenjo B., Ordóñez C., García-Estrada C., Martínez-Valladares M., Balaña-Fouce R. (2019). Current and Promising Novel Drug Candidates against Visceral Leishmaniasis. Pure Appl. Chem..

[34] Sunyoto T., Potet J., Boelaert M. (2018). Why Miltefosine—A Life-Saving Drug for Leishmaniasis—Is Unavailable to People Who Need It the Most. BMJ Glob. Health.

[35] Carnielli J.B.T., Monti-Rocha R., Costa D.L., Molina Sesana A., Pansini L.N.N., Segatto M., Mottram J.C., Costa C.H.N., Carvalho S.F.G., Dietze R. (2019). Natural Resistance of *Leishmania infantum* to Miltefosine Contributes to the Low Efficacy in the Treatment of Visceral Leishmaniasis in Brazil. Am. J. Trop. Med. Hyg..

[36] Lipinski C.A., Lombardo F., Dominy B.W., Feeney P.J. (2012). Experimental and Computational Approaches to Estimate Solubility and Permeability in Drug Discovery and Development Settings. Adv. Drug Deliv. Rev..

[37] Veber D.F., Johnson S.R., Cheng H.-Y., Smith B.R., Ward K.W., Kopple K.D. (2002). Molecular Properties That Influence the Oral Bioavailability of Drug Candidates. J. Med. Chem..

[38] Temraz M.G., Elzahhar P.A., El-Din A., Bekhit A., Bekhit A.A., Labib H.F., Belal A.S.F. (2018). Anti-Leishmanial Click Modifiable Thiosemicarbazones: Design, Synthesis, Biological Evaluation and in Silico Studies. Eur. J. Med. Chem..

[39] Yang Z.-Y., He J.-H., Lu A.-P., Hou T.-J., Cao D.-S. (2020). Application of Negative Design To Design a More Desirable Virtual Screening Library. J. Med. Chem..

[40] Cavalcante-Costa V.S., Queiroz-Oliveira T., Horta M.F., Castro-Gomes T., de Souza W. (2022). Leishmania and Their Vertebrate Host Cells. Lifecycles of Pathogenic Protists in Humans.

[41] Bogdan C., Donhauser N., Döring R., Röllinghoff M., Diefenbach A., Rittig M.G. (2000). Fibroblasts as Host Cells in Latent Leishmaniosis. J. Exp. Med..

[42] Valigurová A., Kolářová I. (2023). Unrevealing Mystery Latent Leishmaniasis: What Cells Can Host Leishmania?. Pathogens.

[43] Vatsadze S.Z., Loginova Y.D., dos Passos Gomes G., Alabugin I.V. (2017). Stereoelectronic Chameleons: The Donor–Acceptor Dichotomy of Functional Groups. Chem.—A Eur. J..

[44] Naderer T., McConville M.J. (2008). The Leishmania–Macrophage Interaction: A Metabolic Perspective. Cell. Microbiol..

[45] López R., Cuca L.E., Delgado G. (2009). Antileishmanial and Immunomodulatory Activity of Xylopia Discreta. Parasite Immunol..

[46] Machín L., Nápoles R., Gille L., Monzote L. (2021). *Leishmania amazonensis* Response to Artemisinin and Derivatives. Parasitol. Int..

[47] Ferrence L.M., Gajula A., Jones M.A. (2023). Surprising Effects of Rocking Motion on *Leishmania tarentolae* Behavior in Culture and Implications for Cell Stress. Stresses.

[48] Iovannisci D.M., Paul Plested C., Moe G.R. (2010). Evidence for Rosettes as an Unrecognized Stage in the Life Cycle of *Leishmania* Parasites. J. Eukaryot. Microbiol..

[49] Fernandes Rodrigues J.C., Souza W. (2008). de Ultrastructural Alterations in Organelles of Parasitic Protozoa Induced by Different Classes of Metabolic Inhibitors. Curr. Pharm. Des..

[50] Kelly F.D., Sanchez M.A., Landfear S.M. (2020). Touching the Surface: Diverse Roles for the Flagellar Membrane in Kinetoplastid Parasites. Microbiol. Mol. Biol. Rev..

[51] Kelly F.D., Yates P.A., Landfear S.M. (2021). Nutrient Sensing in Leishmania: Flagellum and Cytosol. Mol. Microbiol..

[52] Dagger F., Valdivieso E., Marcano A.K., Ayesta C. (2013). Regulatory Volume Decrease in *Leishmania mexicana*: Effect of Anti-Microtubule Drugs. Mem. Inst. Oswaldo Cruz.

[53] Oliveira S.S.C., Marques C.S.F., de Sousa D.P., Andrade L.N., Fricks A.T., Jain S., Branquinha M.H., Souto E.B., Santos A.L.S., Severino P. (2021). Analysis of the Mechanisms of Action of Isopentenyl Caffeate against *Leishmania*. Biochimie.

[54] Antinarelli L.M.R., Midlej V., da Silva E.D.S., Coelho E.A.F., da Silva A.D., Coimbra E.S. (2023). Exploring the Repositioning of the Amodiaquine as Potential Drug against Visceral Leishmaniasis: The in Vitro Effect against *Leishmania infantum* Is Associated with Multiple Mechanisms, Involving Mitochondria Dysfunction, Oxidative Stress and Loss of Cell Cycle Control. Chem.—Biol. Interact..

[55] Korotkov S.M. (2023). Mitochondrial Oxidative Stress Is the General Reason for Apoptosis Induced by Different-Valence Heavy Metals in Cells and Mitochondria. Int. J. Mol. Sci..

[56] Yamamoto E.S., de Jesus J.A., Bezerra-Souza A., Brito J.R., Lago J.H.G., Laurenti M.D., Passero L.F.D. (2020). Tolnaftate Inhibits Ergosterol Production and Impacts Cell Viability of *Leishmania* sp.. Bioorgan. Chem..

[57] de Oliveira Silva Nunes D.C., Costa M.S., Bispo-da-Silva L.B., Ferro E.A.V., Zóia M.A.P., Goulart L.R., Rodrigues R.S., de Melo Rodrigues V., Yoneyama K.A.G. (2022). Mitochondrial Dysfunction on *Leishmania (Leishmania) amazonensis* Induced by Ketoconazole: Insights into Drug Mode of Action. Mem. Inst. Oswaldo Cruz.

[58] Paul A., Roy P.K., Babu N.K., Singh S. (2024). Clotrimazole Causes Membrane Depolarization and Induces Sub G0 Cell Cycle Arrest in *Leishmania donovani*. Acta Trop..

[59] de Macedo-Silva S.T., Visbal G., Souza G.F., dos Santos M.R., Cämmerer S.B., de Souza W., Rodrigues J.C.F. (2022). Benzylamines as Highly Potent Inhibitors of the Sterol Biosynthesis Pathway in *Leishmania amazonensis* Leading to Oxidative Stress and Ultrastructural Alterations. Sci. Rep..

[60] Duszenko M., Ginger M.L., Brennand A., Gualdrón-López M., Colombo M.I., Coombs G.H., Coppens I., Jayabalasingham B., Langsley G., Lisboa de Castro S. (2011). Autophagy in Protists. Autophagy.

[61] Romano P.S., Akematsu T., Besteiro S., Bindschedler A., Carruthers V.B., Chahine Z., Coppens I., Descoteaux A., Lopes Alberto Duque T., He C.Y. (2023). Autophagy in Protists and Their Hosts: When, How and Why?. Autophagy Rep..

[62] Pedra-Rezende Y., Macedo I.S., Midlej V., Mariante R.M., Menna-Barreto R.F.S. (2022). Different Drugs, Same End: Ultrastructural Hallmarks of Autophagy in Pathogenic Protozoa. Front. Microbiol..

[63] Sandes J.M., de Figueiredo R.C.B.Q. (2022). The Endoplasmic Reticulum of Trypanosomatids: An Unrevealed Road for Chemotherapy. Front. Cell. Infect. Microbiol..

[64] Machado P.d.A., Gomes P.S., Midlej V., Coimbra E.S., de Matos Guedes H.L. (2021). PF-429242, a Subtilisin Inhibitor, Is Effective in Vitro Against *Leishmania infantum*. Front. Microbiol..

[65] Menna-Barreto R.F.S. (2019). Cell Death Pathways in Pathogenic Trypanosomatids: Lessons of (over)Kill. Cell Death Dis..

[66] Lamotte S., Späth G.F., Rachidi N., Prina E. (2017). The Enemy within: Targeting Host–Parasite Interaction for Antileishmanial Drug Discovery. PLoS Neglect. Trop. Dis..

[67] Chawla B., Madhubala R. (2010). Drug Targets in *Leishmania*. J. Parasit. Dis..

[68] Beltran-Hortelano I., Alcolea V., Font M., Pérez-Silanes S. (2022). Examination of Multiple *Trypanosoma cruzi* Targets in a New Drug Discovery Approach for Chagas Disease. Bioorgan. Med. Chem..

[69] Jin Y., Basu S., Feng M., Ning Y., Munasinghe I., Joachim A.M., Li J., Madden R., Burks H., Gao P. (2023). CYP5122A1 Encodes an Essential Sterol C4-Methyl Oxidase in *Leishmania donovani* and Determines the Antileishmanial Activity of Antifungal Azoles. Res. Sq..

[70] Pinto Pinheiro M., da Silva Emery F., Cristina Nonato M. (2013). Target Sites for the Design of Anti-Trypanosomatid Drugs Based on the Structure of Dihydroorotate Dehydrogenase. Curr. Pharm. Des..

[71] Battista T., Colotti G., Ilari A., Fiorillo A. (2020). Targeting Trypanothione Reductase, a Key Enzyme in the Redox Trypanosomatid Metabolism, to Develop New Drugs against *Leishmaniasis* and *Trypanosomiases*. Molecules.

[72] Sasidharan S., Saudagar P. (2022). Knockout of Tyrosine Aminotransferase Gene by Homologous Recombination Arrests Growth and Disrupts Redox Homeostasis in *Leishmania* Parasite. Parasitol. Res..

[73] Panecka-Hofman J., Poehner I. (2023). Structure and Dynamics of Pteridine Reductase 1: The Key Phenomena Relevant to Enzyme Function and Drug Design. Eur. Biophys. J..

[74] Kourbeli V., Chontzopoulou E., Moschovou K., Pavlos D., Mavromoustakos T., Papanastasiou I.P. (2021). An Overview on Target-Based Drug Design against Kinetoplastid Protozoan Infections: Human African Trypanosomiasis, Chagas Disease and Leishmaniases. Molecules.

[75] Chaves E.J.F., Padilha I.Q.M., Araújo D.A.M., Rocha G.B. (2018). Determining the Relative Binding Affinity of Ricin Toxin A Inhibitors by Using Molecular Docking and Nonequilibrium Work. J. Chem. Inf. Model..

[76] Guterres H., Im W. (2020). Improving Protein-Ligand Docking Results with High-Throughput Molecular Dynamics Simulations. J. Chem. Inf. Model..

[77] Rocha S.F.L.S., Sant’Anna C.M.R. (2019). A Procedure Combining Molecular Docking and Semiempirical Method PM7 for Identification of Selective Shp2 Inhibitors. Biopolymers.

[78] Ryde U., Söderhjelm P. (2016). Ligand-Binding Affinity Estimates Supported by Quantum-Mechanical Methods. Chem. Rev..

[79] Lepesheva G.I., Friggeri L., Waterman M.R. (2018). CYP51 as Drug Targets for Fungi and Protozoan Parasites: Past, Present and Future. Parasitology.

[80] Lepesheva G.I., Villalta F., Waterman M.R., Weiss L.M., Tanowitz H.B., Kirchhoff L.V. (2011). Targeting *Trypanosoma cruzi* Sterol 14α-Demethylase (CYP51). Advances in Parasitology.

[81] Mansoldo F.R.P., Carta F., Angeli A., Cardoso V.d.S., Supuran C.T., Vermelho A.B. (2020). Chagas Disease: Perspectives on the Past and Present and Challenges in Drug Discovery. Molecules.

[82] Villalta F., Dobish M.C., Nde P.N., Kleshchenko Y.Y., Hargrove T.Y., Johnson C.A., Waterman M.R., Johnston J.N., Lepesheva G.I. (2013). VNI Cures Acute and Chronic Experimental Chagas Disease. J. Infect. Dis..

[83] Guedes-da-Silva F.H., Batista D.d.G.J., Da Silva C.F., Pavão B.P., Batista M.M., Moreira O.C., Souza L.R.Q., Britto C., Rachakonda G., Villalta F. (2019). Successful Aspects of the Coadministration of Sterol 14α-Demethylase Inhibitor VFV and Benznidazole in Experimental Mouse Models of Chagas Disease Caused by the Drug-Resistant Strain of *Trypanosoma cruzi*. ACS Infect. Dis..

[84] Ispikoudi M., Amvrazis M., Kontogiorgis C., Koumbis A.E., Litinas K.E., Hadjipavlou-Litina D., Fylaktakidou K.C. (2010). Convenient Synthesis and Biological Profile of 5-Amino-Substituted 1,2,4-Oxadiazole Derivatives. Eur. J. Med. Chem..

[85] Mosmann T. (1983). Rapid Colorimetric Assay for Cellular Growth and Survival: Application to Proliferation and Cytotoxicity Assays. J. Immunol. Methods.

[86] de Morais-Teixeira E., de Carvalho A.S., da Costa J.C., Duarte S.L., Mendonça J.S., Boechat N., Rabello A. (2008). In Vitro and in Vivo Activity of Meglumine Antimoniate Produced at Farmanguinhos-Fiocruz, Brazil, against *Leishmania* (Leishmania) *Amazonensis*, L (L.) *Chagasi* and L (Viannia) Braziliensis. Mem. Inst. Oswaldo Cruz.

[87] Rondon F.C.M., Bevilaqua C.M.L., Accioly M.P., de Morais S.M., de Andrade-Júnior H.F., de Carvalho C.A., Lima J.C., Magalhães H.C.R. (2012). In Vitro Efficacy of Coriandrum Sativum, Lippia Sidoides and Copaifera Reticulata against *Leishmania chagasi*. Rev. Bras. Parasitol. Vet..

[88] Holanda V.N., da Silva W.V., do Nascimento P.H., Silva S.R.B., Cabral Filho P.E., Assis S.P.d.O., da Silva C.A., de Oliveira R.N., de Figueiredo R.C.B.Q., Lima V.L.d.M. (2020). Antileishmanial Activity of 4-Phenyl-1-[2-(Phthalimido-2-Yl)Ethyl]-1H-1,2,3-Triazole (PT4) Derivative on *Leishmania amazonensis* and *Leishmania braziliensis*: In Silico ADMET, in Vitro Activity, Docking and Molecular Dynamic Simulations. Bioorgan. Chem..

[89] Hargrove T.Y., Wawrzak Z., Liu J., Nes W.D., Waterman M.R., Lepesheva G.I. (2011). Substrate Preferences and Catalytic Parameters Determined by Structural Characteristics of Sterol 14α-Demethylase (CYP51) from *Leishmania infantum*. J. Biol. Chem..

[90] Moreno M.A., Abramov A., Abendroth J., Alonso A., Zhang S., Alcolea P.J., Edwards T., Lorimer D., Myler P.J., Larraga V. (2014). Structure of Tyrosine Aminotransferase from *Leishmania infantum*. Acta Crystall. Sect. F.

[91] Ilari A., Baiocco P., Messori L., Fiorillo A., Boffi A., Gramiccia M., Di Muccio T., Colotti G. (2012). A Gold-Containing Drug against Parasitic Polyamine Metabolism: The X-Ray Structure of Trypanothione Reductase from *Leishmania infantum* in Complex with Auranofin Reveals a Dual Mechanism of Enzyme Inhibition. Amino Acids.

[92] Anandakrishnan R., Aguilar B., Onufriev A.V. (2012). H++ 3.0: Automating pK Prediction and the Preparation of Biomolecular Structures for Atomistic Molecular Modeling and Simulations. Nucleic Acids Res..

[93] Phillips J.C., Hardy D.J., Maia J.D.C., Stone J.E., Ribeiro J.V., Bernardi R.C., Buch R., Fiorin G., Hénin J., Jiang W. (2020). Scalable Molecular Dynamics on CPU and GPU Architectures with NAMD. J. Chem. Phys..

[94] Jones G., Willett P., Glen R.C., Leach A.R., Taylor R. (1997). Development and Validation of a Genetic Algorithm for Flexible Docking. J. Mol. Biol..

[95] Tian C., Kasavajhala K., Belfon K.A.A., Raguette L., Huang H., Migues A.N., Bickel J., Wang Y., Pincay J., Wu Q. (2020). ff19SB: Amino-Acid-Specific Protein Backbone Parameters Trained against Quantum Mechanics Energy Surfaces in Solution. J. Chem. Theory Comput..

[96] Vassetti D., Pagliai M., Procacci P. (2019). Assessment of GAFF2 and OPLS-AA General Force Fields in Combination with the Water Models TIP3P, SPCE, and OPC3 for the Solvation Free Energy of Druglike Organic Molecules. J. Chem. Theory Comput..

[97] Roe D.R., Cheatham T.E.I. (2013). PTRAJ and CPPTRAJ: Software for Processing and Analysis of Molecular Dynamics Trajectory Data. J. Chem. Theory Comput..

[98] Baek M., DiMaio F., Anishchenko I., Dauparas J., Ovchinnikov S., Lee G.R., Wang J., Cong Q., Kinch L.N., Schaeffer R.D. (2021). Accurate prediction of protein structures and interactions using a three-track neural network. Science.

