# Supplementary material for: 1,2,4-Oxadiazole Derivatives: Physicochemical Properties, Antileishmanial Potential, Docking and Molecular Dynamic Simulations of Leishmania infantum Target Proteins
Source: Molecules. 2024 Sep 30;29(19):4654. doi: 10.3390/molecules29194654 (PMC11478322; doi:10.3390/molecules29194654)
Supplement: Supplementary file 1 [file molecules-29-04654-s001.zip › Supplementary Material.pdf]

1,2,4-Oxadiazole derivatives: Physicochemical properties, antileishmanial potential and docking and molecular dynamic simulations of *Leishmania infantum* target proteins

Deyzi Barbosa[1], Vanderlan Holanda[2], Elton Lima[3], Marton Cavalcante[4], Maria Carolina Castro[5], Elton Chaves[6], Gerd Rocha[6], Carla Silva[7], Ronaldo Oliveira[8] and Regina Figueiredo[1,] *

[1] Department of Microbiology, Aggeu Magalhães Institute (IAM-FIOCRUZ), 50740-465, Recife, PE, Brazil; deyzi-caroline@hotmail.com (D.B.); regina.bressan@fiocruz.br (R.F.)
[2] University Center of Vitória de Santo Antão (UNIVISA), 55610-050, Vitória de Santo Antão, PE, Brazil; vanderlan.nogueira@gmail.com
[3] Center for Exact and Natural Sciences, Federal University of Pernambuco (UFPE), 50740-560, Recife, PE, Brazil; eltonmarlon@gmail.com
[4] Federal University of Pernambuco (UFPE), 50670-420, Recife, PE, Brazil; m.kaiquecavalcante@hotmail.com
[5] Department of Immunology, Aggeu Magalhães Institute (IAM-FIOCRUZ), 50740-465, Recife, PE, Brazil; mariacarolinawanderley@gmail.com
[6] Department of Chemistry, Federal University of Paraíba (UFPB), 58051-900, João Pessoa, PB, Brazil; chavesejf@gmail.com (E.C.); gbr@quimica.ufpb.br (G.R.)
[7] Department of Fundamental Chemistry, Federal University of Pernambuco (UFPE), 50740-540, Recife, PE, Brazil; cjasmine0803@gmail.com
[8] Department of Chemistry, Federal Rural University of Pernambuco (UFRPE), 52171-900, Recife, PE, Brazil; ronaldo.noliveira@ufrpe.br
* Correspondence: regina.bressan@fiocruz.br

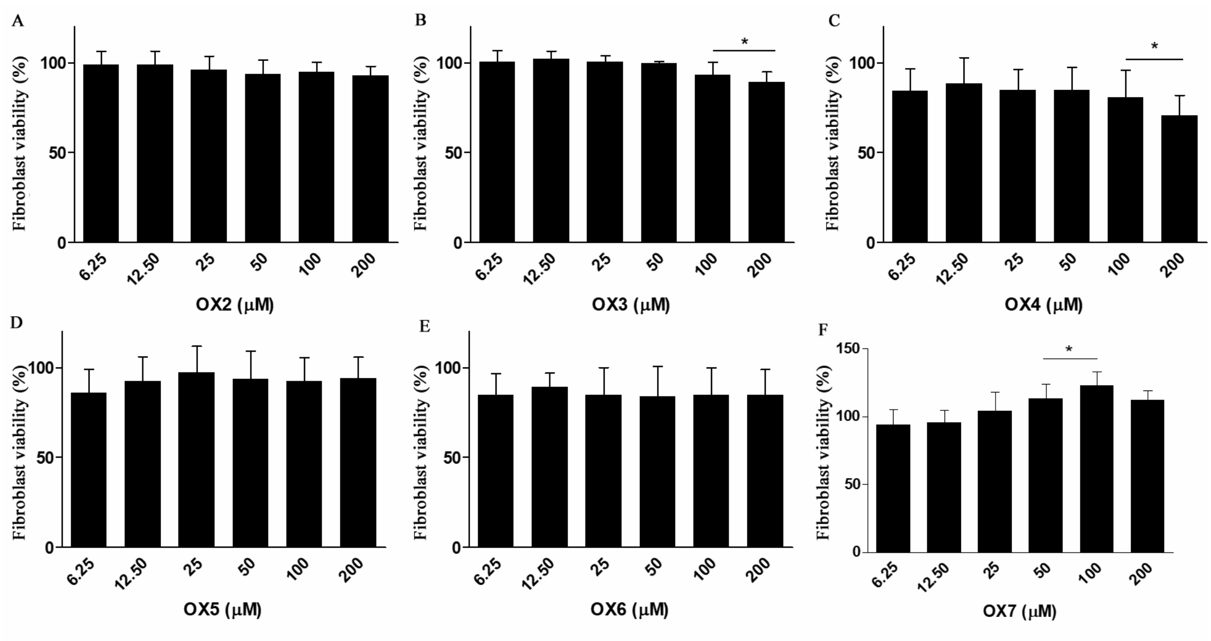

Figure S1. Effects of **Ox2-Ox7** on the viability of Fibroblasts (L929) after 48 hours of treatment. Values represent the mean ± standard deviation of three independent experiments performed in triplicate. *Significant differences at $p < 0.05$ compared to the untreated control.

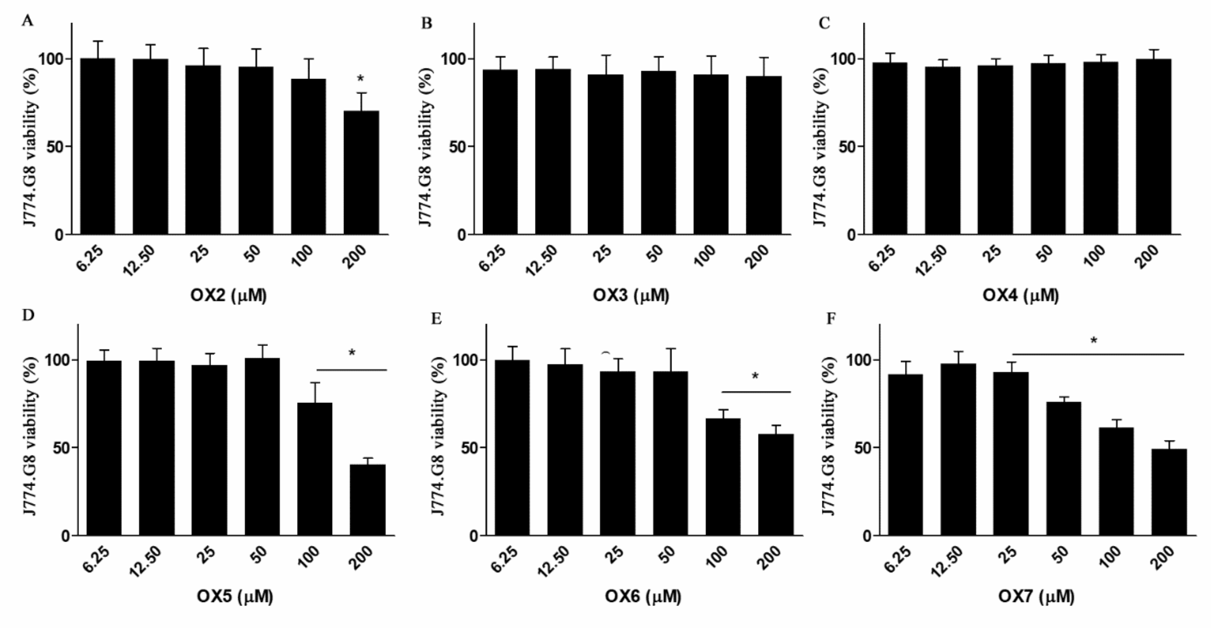

Figure S2. Effects of **Ox2-Ox7** on the viability of macrophages (J774.G8) after 48 hours of treatment. Values represent the mean ± standard deviation of three independent experiments performed in triplicate. *Significant differences at $p < 0.05$ compared to the untreated control.

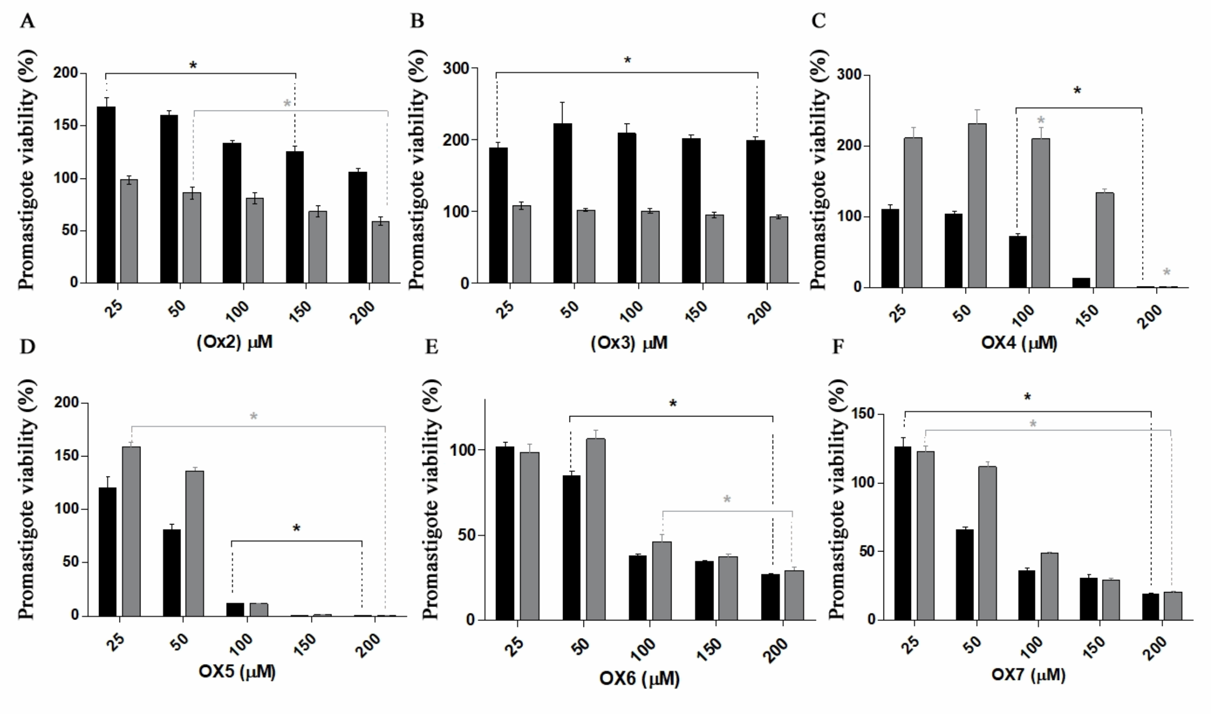

Figure S3. Effects of **Ox2-Ox7** on *L. infantum* promastigotes after 24 and 48 hours of treatment. Values represent the mean ± standard deviation of three independent experiments performed in triplicate. *Significant difference compared to the untreated control group, p <0.05.

Homology Modeling

The X-ray structures from the PDB 3C61 and 1W0C were used as templates to predict the structures of dihydroorotate reductase (UniProt ID A0A6L0WWX1) and pteridine reductase (UniProt ID A4I067) from *L. infantum*, respectively, in the RoseTTAFold software [1]. To recover the structure of the cofactors, the resulting structure (predicted 3D structure) was aligned according to its template, and the coordinates of the cofactor were merged into the modeled structure. The resulting geometries from the homology modeling protocol and those obtained from the Protein Data Bank were optimized before the molecular docking procedure. The 3D structures of the receptors were parameterized by using the ff19SB force field [2], and cofactors were parameterized with the GAFF2 force field [3]. Regarding the parameters of the heme group from the sterol 14-methyltransferase, we used the parameters obtained in the work of Ryde and co-workers. The resulting geometries were relaxed considering an implicit solvent model over 20,000 conjugate gradient steps in the NAMD software [4].

MD Trajectory Analysis

The analysis of molecular dynamics trajectory was carried out by using CPPTRAJ software (version 6.18.1) [5]. The following analysis was performed:

(i) **Root Mean Square Deviation (RMSD).** RMSD calculations were performed for the backbone atoms of the protein (i.e.: C, Cα, N, and O) and the heavy atoms of the **Ox1** compound, which were aligned according to the first frame of the trajectory before RMSD calculation.

(ii) **Interaction energy.** Electrostatic and van der Waals (vdW) interactions were calculated for all residues around the **Ox1** compound throughout the last 100 ns of MD simulation; a separate electrostatic and van der Waals cutoff was applied, 12 Å for both.

(iii) **Centre of mass distance analysis.** The distance between the center of mass of the protein and **Ox1** was calculated to define the local stability of the ligand in the binding site, since abrupt displacements may be related to unspecificity concerning the binding site.

(iii) **Molecular mechanics with generalized Born and Surface Area** (**MM/GBSA**)**.** The MM/GBSA method was used to predict the binding free energy of **Ox1** complexes. For this, 25 frames from the last 50 ns of MD simulation were used. The MMPBSA.py script available in the AMBERTOOLS package was used in default mode.

(iv) **Enthalpy of binding.** Single-point quantum chemistry calculations were employed to measure relative quantities of the binding enthalpy (ΔH_{bind}) for all simulated complexes, using PM7, a semiempirical quantum chemical method (SQM). The SQM parameters were set according to the study of Urquiza-Carvalho and co-workers: (i) linear scaling algorithm MOZYME, available in the MOPAC package; (ii) SCF convergence criteria in default configuration and cutoff radius of 9 A; (iii) COSMO implicit solvent field with a relative permittivity of 78.4; (v) and an effective solvent molecule radius of 1.3 A. Such calculations were performed for different frames of the trajectory from the time range of 50 to 100 ns (every 200 ps); **Eq. 1** was used to calculate the binding enthalpy and averages, as well as standard deviations were calculated using 25 frames from the last 50 ns of the MD simulation.

$$\Delta H_{bind} \; \Delta H_f^{complex} - \left(\Delta H_f^{ligand} + \Delta H_f^{receptor}\right)$$

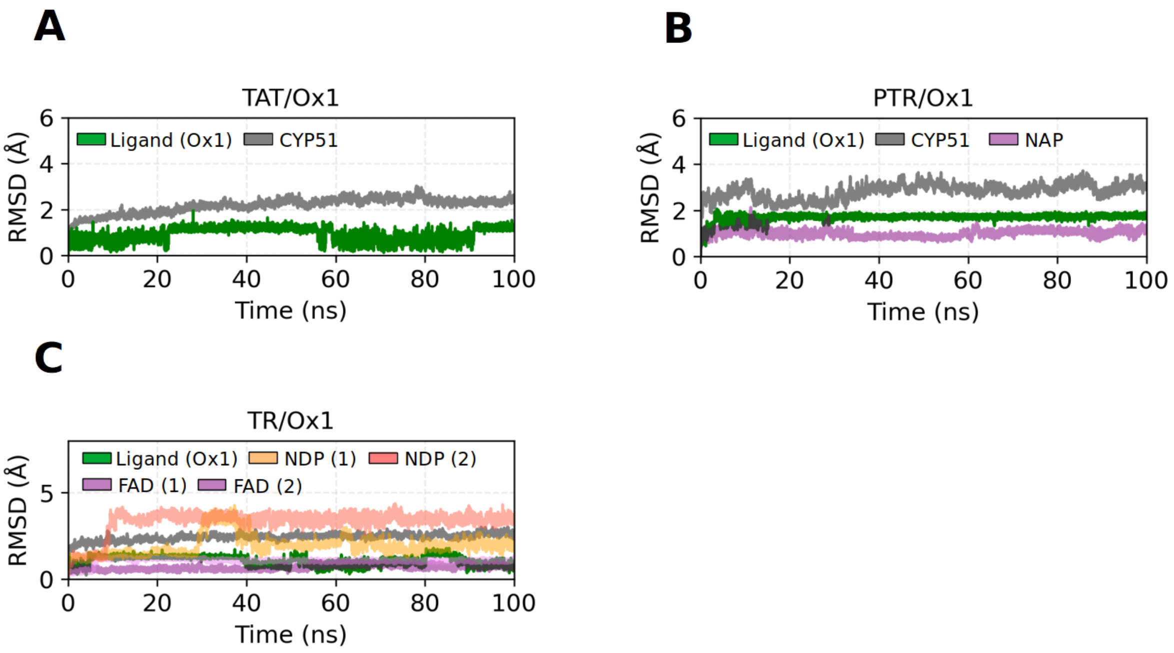

Figure S4. RMSD profile of the MD trajectories of the complexes **TAT/Ox1** (**A**), **PTR1/Ox1** (**B**), and **TR/Ox1** (**C**).

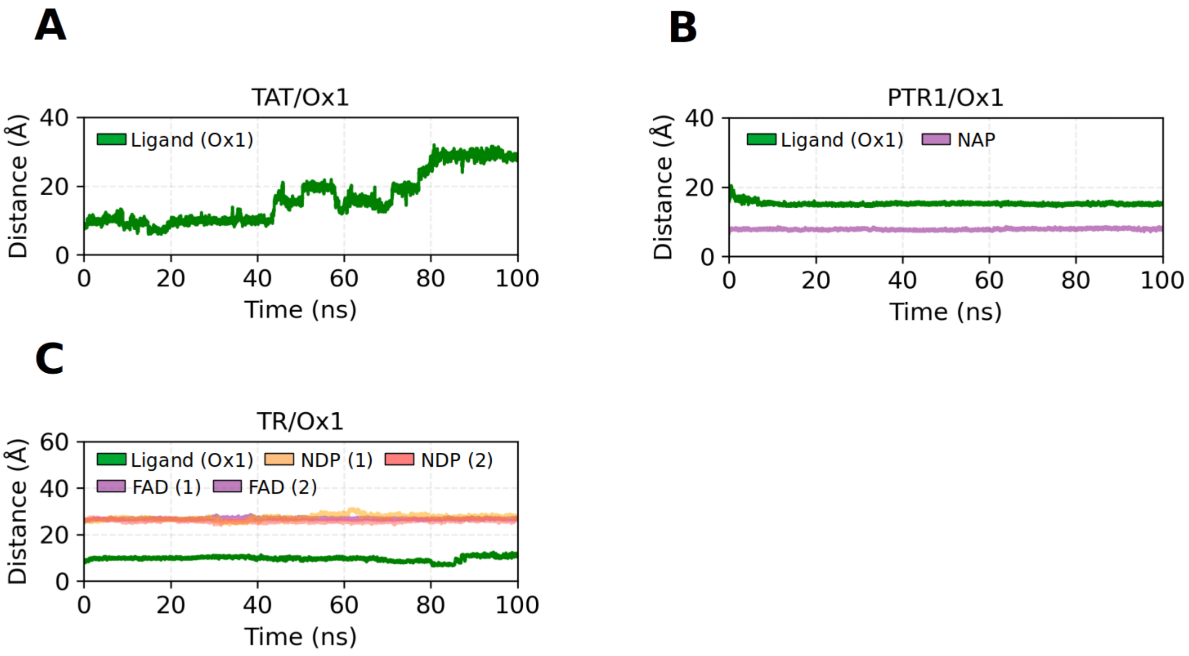

Figure S5. Center of mass distance analysis of the MD trajectories of the complexes **TAT/Ox1** (**A**), **PTR1/Ox1** (**B**), and **TR/Ox1** (**C**).

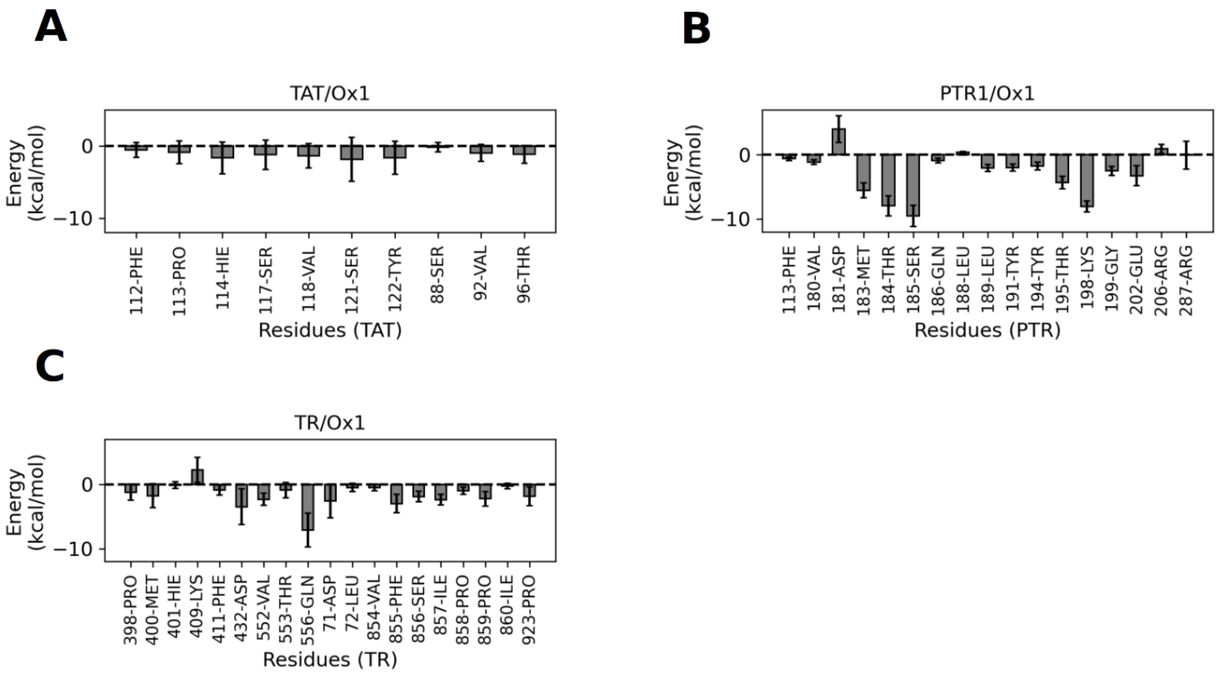

Figure S6. Interaction energy profile of the MD trajectories of the complexes **TAT/Ox1** (**A**), **PTR1/Ox1** (**B**), and **TR/Ox1** (**C**).

Table S1. Hydrogen bonds profile between the Ox1 compound and molecular targets of *L. infantum* during MD simulation. The last column comprises the occurrence (total of frames during MD simulation).

Receptor	Acceptor		Donor		Percentage (%)
	**Residue/Ligand**	**Atom**	**Residue/Ligand**	**Atom**	
CYP51	Ox1	N1	Tyr74	OH@HH	87.72
	Met329	O	Ox1	N@H	96.68
TAT	-	-	-	-	-
PTR1	Ox1	N1	Ser185	N@H	96.00
	Ser185	OG	Ox1	N@H	93.16
TR	Ox1	N1	Gln556	NE2@HE21	26.80
	Gln556	OE1	Ox	N@H	12.68

Video S1: RMSD Profile of Ox1 at the CYP51 Binding Site Demonstrates Structural Stability Over 100 ns Interval.

REFERENCES (SI)

[1] M. Baek, F. DiMaio, I. Anishchenko, J. Dauparas, S. Ovchinnikov, G.R. Lee, J. Wang, Q. Cong, L.N. Kinch, R.D. Schaeffer, C. Millán, H. Park, C. Adams, C.R. Glassman, A. DeGiovanni, J.H. Pereira, A.V. Rodrigues, A.A. van Dijk, A.C. Ebrecht, D.J. Opperman, T. Sagmeister, C. Buhlheller, T. Pavkov-Keller, M.K. Rathinaswamy, U. Dalwadi, C.K. Yip, J.E. Burke, K.C. Garcia, N.V. Grishin, P.D. Adams, R.J. Read, D. Baker, Accurate prediction of protein structures and interactions using a three-track neural network, Science 373 (2021) 871–876. https://doi.org/10.1126/science.abj8754.

[2] C. Tian, K. Kasavajhala, K.A.A. Belfon, L. Raguette, H. Huang, A.N. Migues, J. Bickel, Y. Wang, J. Pincay, Q. Wu, C. Simmerling, ff19SB: Amino-Acid-Specific Protein Backbone Parameters Trained against Quantum Mechanics Energy Surfaces in Solution, J. Chem. Theory Comput. 16 (2020) 528–552. https://doi.org/10.1021/acs.jctc.9b00591.

[3] D. Vassetti, M. Pagliai, P. Procacci, Assessment of GAFF2 and OPLS-AA General Force Fields in Combination with the Water Models TIP3P, SPCE, and OPC3 for the Solvation Free Energy of Druglike Organic Molecules, J. Chem. Theory Comput. 15 (2019) 1983–1995. https://doi.org/10.1021/acs.jctc.8b01039.

[4] J.C. Phillips, D.J. Hardy, J.D.C. Maia, J.E. Stone, J.V. Ribeiro, R.C. Bernardi, R. Buch, G. Fiorin, J. Hénin, W. Jiang, R. McGreevy, M.C.R. Melo, B.K. Radak, R.D. Skeel, A. Singharoy, Y. Wang, B. Roux, A. Aksimentiev, Z. Luthey-Schulten, L.V. Kalé, K. Schulten, C. Chipot, E. Tajkhorshid, Scalable molecular dynamics on CPU and GPU architectures with NAMD, J. Chem. Phys. 153 (2020) 044130. https://doi.org/10.1063/5.0014475.

[5] D.R. Roe, T.E.I. Cheatham, PTRAJ and CPPTRAJ: Software for Processing and Analysis of Molecular Dynamics Trajectory Data, J. Chem. Theory Comput. 9 (2013) 3084–3095. https://doi.org/10.1021/ct400341p.
